# Metabolic alterations in urine among the patients with severe fever with thrombocytopenia syndrome

**DOI:** 10.1186/s12985-024-02285-2

**Published:** 2024-01-08

**Authors:** Shan-Shan Zhang, Xin Yang, Wan-Xue Zhang, Yiguo Zhou, Ting-Ting Wei, Ning Cui, Juan Du, Wei Liu, Qing-Bin Lu

**Affiliations:** 1 Department of Epidemiology and Biostatistics, School of Public Health, Peking University, Beijing, China; 2 State Key Laboratory of Pathogen and Biosecurity, Beijing Institute of Microbiology and Epidemiology, Beijing, China; 3 Center for Infectious Disease and Policy Research & Global Health and Infectious Diseases Group, Peking University, Beijing, China; 4 Department of Health Policy and Management, School of Public Health, Peking University, Beijing, China; 5 Department of Laboratorial of Science and Technology & Vaccine Research Center, School of Public Health, Peking University, No. 38 Xue-Yuan Road, Haidian District, Beijing, 100191 China; 6 Department of Infectious Diseases, The 154th Hospital, Xinyang, China; 7 Key Laboratory of Epidemiology of Major Diseases (Peking University), Ministry of Education, Beijing, China

**Keywords:** Severe fever with thrombocytopenia syndrome; Urine; Metabolomics; Fatal; Pathogenesis

## Abstract

**Background:**

The pathogenesis of severe fever with thrombocytopenia syndrome (SFTS) remained unclear. We aimed to profile the metabolic alterations in urine of SFTS patients and provide new evidence for its pathogenesis.

**Methods:**

A case–control study was conducted in the 154th hospital in China. Totally 88 cases and 22 controls aged ≥ 18 years were enrolled. The cases were selected from laboratory-confirmed SFTS patients. The controls were selected among SFTSV-negative population. Those with diabetes, cancer, hepatitis and other sexually transmitted diseases were excluded in both groups. Fatal cases and survival cases were 1:1 matched. Inter-group differential metabolites and pathways were obtained, and the inter-group discrimination ability was evaluated.

**Results:**

Tryptophan metabolism and phenylalanine metabolism were the top one important metabolism pathway in differentiating the control and case groups, and the survival and fatal groups, respectively. The significant increase of differential metabolites in tryptophan metabolism, including 5-hydroxyindoleacetate (5-HIAA), L-kynurenine (KYN), 5-hydroxy-L-tryptophan (5-HTP), 3-hydroxyanthranilic acid (3-HAA), and the increase of phenylpyruvic acid and decrease of hippuric acid in phenylalanine metabolism indicated the potential metabolic alterations in SFTSV infection. The increase of 5-HIAA, KYN, 5-HTP, phenylpyruvic acid and hippuric acid were involved in the fatal progress of SFTS patients.

**Conclusions:**

Tryptophan metabolism and phenylalanine metabolism might be involved in the pathogenesis of SFTSV infection. These findings provided new evidence for the pathogenesis and treatment of SFTS.

**Supplementary Information:**

The online version contains supplementary material available at 10.1186/s12985-024-02285-2.

## Background

Severe fever with thrombocytopenia syndrome (SFTS), an emerging tick-borne zoonosis characterized by leukopenia and thrombocytopenia, is caused by SFTS virus (SFTSV), a novel *Bandavirus* of the *Phenuiviridae* family, *Dabie bandavirus* [1]. SFTSV infection presents as mild in most cases but can develop into life-threatening illness and die from multiple organ failure, which occurs in over 10% of the patients [2, 3]. SFTS has been reported mainly in China, Japan, South Korea and Vietnam and included into the list of infectious diseases of priority concern by World Health Organization in 2017 [4, 5].

Studies have shown that SFTSV infection can trigger systemic inflammatory cytokine storms, change the function and proportion of lymphocytes and promote the rapid replication of the virus by inhibiting the host immune system and immune response in various ways [6, 7]. The association of phagocyte phagocytosis and the relevant mechanism of thrombocytopenia has also been investigated in the mouse model [8]. Evidence about pathogenic mechanism of SFTS accumulated though, such studies mostly focused on cell and animal levels and the mechanism of SFTSV pathogenic mechanism remained unclear. It is urgent to elucidate the pathogenesis of SFTS and promote its treatment and prevention.

Metabolomics has been applied to the research of viral and bacterial infectious diseases, such as tuberculosis, hepatitis and human immunodeficiency virus (HIV) [9]. Especially in recent years of the coronavirus disease 2019 (COVID-19) pandemic, metabolomic research has provided plenty of evidence for the pathogenesis of severe acute respiratory syndrome coronavirus 2 (SARS-CoV-2) [10]. Based on the fact that urine can reflect metabolic disorders and provide a novel approach to discover new noninvasive biomarkers in the pathology or treatment of infectious diseases, many studies analyzed urinary metabolic profile of COVID-19 patients, offering unique insights into the pathogenesis of infectious diseases [11, 12].

Metabolomics also plays an important role in exploring the pathogenesis of SFTSV. Li et al. revealed that abnormal arginine metabolism was significantly related to various key clinicopathological changes in SFTS patients [13]. The serum arginine has biological effects on immune regulation, platelet inhibition and vascular endothelial stability regulation, which indicated arginine and its related metabolites might be potential biomarkers for prognosis of SFTS patients and adjuvant therapy drugs [13]. Based on 16S ribosomal RNA sequencing and non-targeted metabolomics method, Xie et al. found that *Akkermansia muciniphila* and its metabolite harmaline can inhibit systemic inflammatory responses through the transmembrane G-protein-coupled receptor-5 (TGR5)-NF-κB signaling, alleviating the severity and mortality of SFTS [14]. All the current evidence based on metabolomics; however, no studies have been conducted to detect the changes of urinary metabolites in SFTS patients to dig the potential mechanism through metabolomics.

Therefore, we investigated the metabolic alterations of urinary metabolites in patients with SFTSV infection and the urinary metabolic differences between fatal and survival patients to further elucidate the pathogenesis of SFTSV infection, and then to provide underlying targets for clinical treatment of SFTS patients based on the pathogenesis of SFTSV.

## Methods

### Study design and participants

A case–control study was conducted in the 154th hospital, the largest sentinel hospital for SFTS treatment in China, locat in Xinyang city, Henan province. All the cases were hospitalized SFTS patients during 2018–2021 and confirmed with SFTSV infection according to guideline released by National Health Commission of China. The cases were selected from laboratory confirmed SFTS patients aged ≥ 18 years, with those who had diabetes, cancer, hepatitis, HIV infection, syphilis and other sexually transmitted diseases excluded. The controls were selected at a 1:4 ratio of cases among SFTSV-negative healthy physical examination people aged ≥ 18 years and those with diabetes, cancer, hepatitis, HIV infection, syphilis and other sexually transmitted diseases were excluded.

Among all the cases, fatal cases and survival cases were 1:1 matched by age (± 3 years), favipiravir therapy (identical) and the interval from symptom onset to sample collection (± 1 day). The outcome of death was obtained by medical record and following up the patients after discharge from hospitalization.

### Information collection and laboratory tests

Demographic and clinical information including individual treatment details were extracted from the medical records by a group of trained physicians. The blood samples were collected from laboratory confirmed patients on admission into the hospital and during the hospitalization. The detection of laboratory indexes was performed every two or three days after the admission to hospital. The laboratory confirmation of SFTSV infection was made according to the criteria as previously described, i.e., positive detection of SFTSV RNA from conventional, nested polymerase chain reaction (PCR) or real-time PCR in the blood of cases [15]. The biochemical indexes were obtained from the examination reports of the hospital and six hematologic indexes were included, which were listed in Additional file 1: Table S1.

### Urine sample collection and metabolites extraction

The urine samples of the case group were collected during the acute phase which was 4–11 days from symptom onset of the SFTS patients. The urine samples of the control group were collected only once in the corresponding same time as the case group. The urine samples were stored at -80°C. The sample was thawed in ice water bath and sampled in proportion according to the osmotic pressure. Water was replenished to 100 μL. After the addition of 400 μL of extract solution (methanol: acetonitrile = 1:1, containing isotopically-labelled internal standard mixture), the samples were vortexed for 30 s, sonicated for 10 min in ice water bath, and incubated for 1 h at − 40 °C to precipitate proteins. Then the sample was centrifuged at 13,800 g for 15 min at 4 °C. The resulting supernatant was transferred to a fresh glass vial for analysis. The quality control sample was prepared by mixing an equal aliquot of the supernatants from all the samples.

### LC–MS analyses

LC–MS/MS analyses were conducted using an UHPLC system (Vanquish, Thermo Fisher Scientific) with a UPLC HSS T3 column (2.1 mm × 100 mm, 1.8 μm) coupled to Orbitrap Exploris 120 mass spectrometer (Orbitrap MS, Thermo). The mobile phase included 5 mmol/L ammonium acetate and 5 mmol/L acetic acid in water (A) and acetonitrile (B). The auto-sampler temperature was 4 °C, and the injection volume was 2 μL. The Orbitrap Exploris 120 mass spectrometer was used to acquire MS/MS spectra on information-dependent acquisition (IDA) mode in the control of the acquisition software (Xcalibur, Thermo). In this mode, the acquisition software evaluates the full scan MS spectrum continuously. The ESI source conditions were set as follows: sheath gas flow rate as 50 Arb, Aux gas flow rate as 15 Arb, capillary temperature 320 °C, full MS resolution as 60,000, MS/MS resolution as 15,000 collision energy as 10/30/60 in NCE mode, spray Voltage as 3.8 kV (positive) or -3.4 kV (negative), respectively. The raw data were converted to the mzXML format for peak detection, extraction, alignment, and integration, using ProteoWizard and processed with an in-house program, which was developed using R and based on XCMS. Then an in-house MS2 database was applied to metabolite annotation, for which the cutoff was set at 0.3.

### Quasi-targeted metabolomics of serum samples

The serum samples were independent from the subjects that was included to obtain the urine samples. Also, the blood samples were collected from laboratory-confirmed patients on admission into the hospital and during the hospitalization. The detailed methods were in supplementary materials.

### Statistical analysis

Continuous variable was expressed as median and interquartile range (IQR), and the comparison was performed using nonparametric Mann–Whitney U test. Categorical variables were described as counts and percentages and compared with χ^2^ test or Fisher’s exact test. The students’ t test was performed for the univariate analysis of urine metabolites. The multivariate analysis for differential urine metabolites screening were conducted by orthogonal partial least square discriminate analysis (OPLS-DA), using software SIMCA (version 14.1, Sartorius Stedim Biotech, Umea, Sweden), in which significant differential metabolites between the two groups were selected according to the variable importance in projection (VIP) values, *P* value (corrected by false discovery rate) and fold change (FC) (VIP ≥ 1, *P* < 0.05, FC > 1 or FC < 1). The enrichment analysis was based on MetaboAnalyst 5.0 (https://www.metaboanalyst.ca/) and the differential pathway was searched on the Kyoto Encyclopedia of Genes and Genomes database (KEGG; https://www.genome.jp/kegg/pathway.html) (*P* < 0.05). The receiver operator characteristic (ROC) curve was conducted and the area under the curve (AUC) was calculated to identify the predictive ability to distinguish between groups. All the statistical analyses and visualization were performed using R version 4.1.2 (R Foundation for Statistical Computing, Vienna, Austria) and STATA 17 (StataCorp LLC, College Station, TX77845, USA). A two-sided *P* < 0.05 was statistically significant.

## Results

### Demographic and clinical manifestations

A total of 88 cases and 22 controls were enrolled in the study, among which there were 44 fatal and 44 survival cases (Tables 1 and 2). The age and the sex composition were all comparable between the case and control groups, as well as between the survival and fatal groups (both *P* > 0.05). No significant differences were observed in the frequencies of hypertension (*P* = 0.550). Among the 88 patients, the top five symptoms and signs of the cases were anorexia (96.6%), feeble (84.0%), nausea (84.1%), Myalgia (70.5%) and fever (69.3%). The age, sex, the interval from symptom onset to admission, the interval from symptom onset to urine sample collection, the frequencies of hypertension, general symptoms and most of the system-specific symptoms were comparable (all *P* > 0.05), except neurological symptoms (*P* < 0.001).

**Table 1:** Demographics and clinical characteristics of SFTS patients and controls in the current study

Characteristics	Total (N = 110)	Controls (n = 22)	Patients (n = 88)	*P*
Age, years, median (IQR)	73 (68–77)	72 (66–77)	73 (68–77)	0.318
Sex, male, n (%)	55 (50.0)	12 (54.5)	43 (48.9)	0.634
Hypertension, n (%)	39 (35.5)	9 (40.9)	30 (34.1)	0.550

*IQR* interquartile range, *SFTS* Severe fever with thrombocytopenia syndrome

**Table 2:** Demographics and clinical characteristics of SFTS patients in the current study

Characteristics	Total (n = 88)	Survival (n = 44)	Fatal (n = 44)	*P*
Age, years, median (IQR)	73 (68–77)	73 (69–78)	73 (68–77)	0.802
Sex, male, n (%)	43 (48.9)	21 (47.7)	22 (50.0)	0.831
The interval from symptom onset to admission, days, median (IQR)	5 (4–5)	5 (4–6)	5 (4–5)	0.737
The interval from symptom onset to urine sample collection, days, median (IQR)	6 (5–8)	7 (5–8)	6 (5–8)	0.818
Hypertension, n (%)	30 (34.1)	18 (40.9)	12 (27.3)	0.177
*General symptoms, n (%)*	88 (100.0)	88 (100.0)	88 (100.0)	1.000
Fever	61 (69.3)	31 (70.5)	30 (68.2)	0.817
Chills	20 (22.7)	8 (18.2)	12 (27.3)	0.309
Headache	14 (15.9)	6 (13.6)	8 (18.2)	0.560
Dizziness	24 (27.3)	10 (22.7)	14 (31.8)	0.338
Feeble	84 (95.5)	43 (97.7)	41 (93.2)	0.609
Myalgia	62 (70.5)	33 (75.0)	29 (65.9)	0.350
Lymphadenectasis	59 (67.0)	27 (61.4)	32 (72.7)	0.257
*Gastrointestinal symptoms, n (%)*	87 (98.9)	43 (97.7)	44 (100.0)	1.000
Anorexia	85 (96.6)	42 (95.5)	43 (97.7)	1.000
Nausea	74 (84.1)	36 (81.8)	38 (86.4)	0.560
Vomit	29 (33.0)	14 (31.8)	15 (34.1)	0.821
Diarrhea	44 (50.0)	15 (34.1)	29 (65.9)	0.003
*Bleeding symptoms, n (%)*	19 (21.6)	7 (15.9)	12 (27.3)	0.195
Melena	7 (8.0)	2 (4.5)	5 (11.4)	0.431
Hemoptysis	6 (6.8)	4 (9.1)	2 (4.5)	0.672
Haematemesis	3 (3.4)	1 (2.3)	2 (4.5)	1.000
Hematuria	8 (9.1)	1 (2.3)	7 (15.9)	0.064
Petechial	0	0	0	1.000
*Neurological symptoms, n (%)*	52 (59.1)	17 (38.6)	35 (79.5)	< 0.001
Dysphoric	26 (29.5)	4 (9.1)	22 (50.0)	< 0.001
Convulsion	10 (11.4)	5 (11.4)	5 (11.4)	1.000
Blurred Mind	27 (30.7)	4 (9.1)	23 (52.3)	< 0.001
Coma/Somnolence	41 (46.6)	14 (31.8)	27 (61.4)	0.005

*IQR* interquartile range, *SFTS* Severe fever with thrombocytopenia syndrome

### Differential urinary metabolites between the case and control groups

**Fig. 1:**
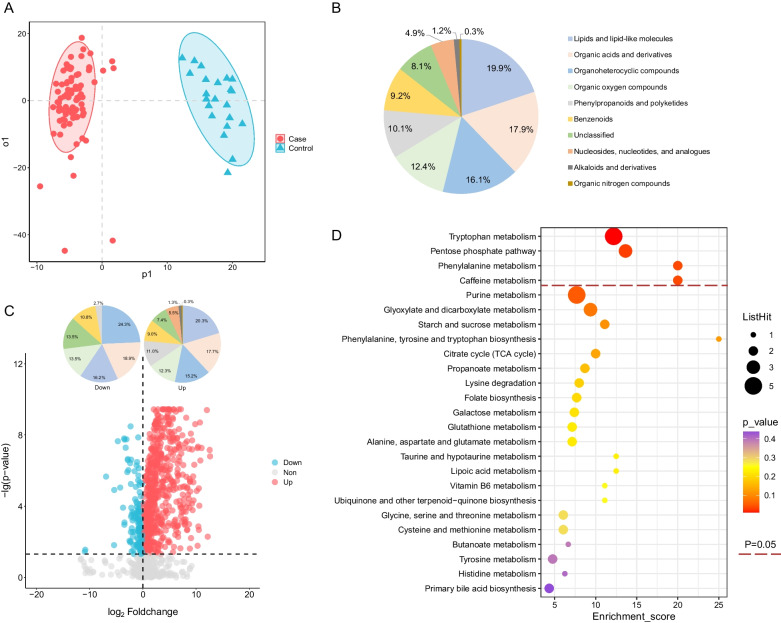
Differential urinary metabolites and metabolic pathways between the case and control groups. **A** The OPLS-DA model of the two groups. **B** The super classes of significant differential urinary metabolites between the two groups. **C** The volcano plot and super classes of significantly up-regulated and down-regulated urinary metabolites of the cases in contrast to the controls. These two pie charts share the same legends with Panel B. **D** The KEGG pathway mapping of significant differential urinary metabolites between the patients and healthy controls. KEGG, Kyoto Encyclopedia of Genes and Genomes database. OPLS-DA orthogonal partial least square discriminate analysis

A clear clustering of cases was obtained using the OPLS-DA model, in which samples between the case and control groups were separated obviously (Fig. 1A). A total of 347 metabolites were differentiated from the two groups, and classified into super classes including lipids and lipid-like molecules, organic acids and derivatives (19.9%), organoheterocyclic compounds (17.9%), organic oxygen compounds (16.1%), phenylpropanoids and polyketides (10.1%), benzenoids (9.2%), nucleosides, nucleotides, and analogues (4.9%), alkaloids and derivatives (1.2%), and organic nitrogen compounds (0.3%) (Fig. 1B). Among all the differential urinary metabolites, there were 310 up-regulated and 37 down-regulated. The top three super classes of down-regulated and up-regulated metabolites were organoheterocyclic compounds (down: 24.3%, up: 15.2%), organic acids and derivatives (down: 18.9%, up: 17.7%), and lipids and lipid-like molecules (down: 16.2%, up: 20.3%) (Fig. 1C).

### Metabolic pathways between the case and control groups

**Fig. 2:**
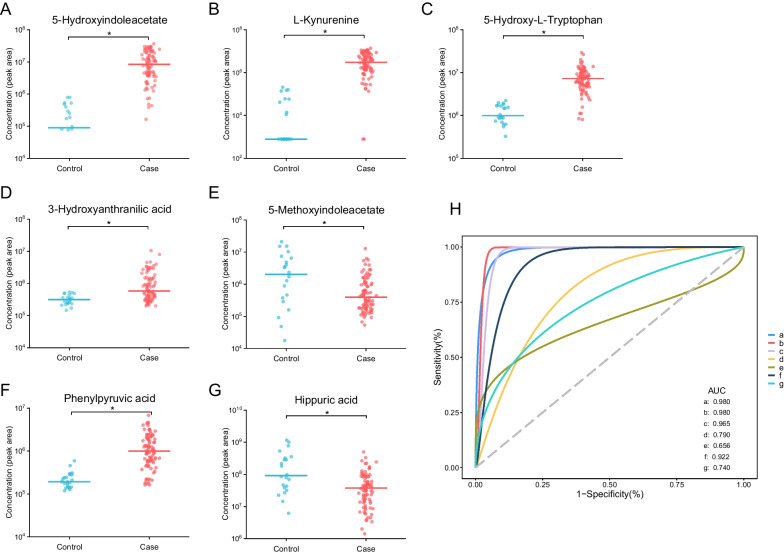
The concentration comparison of the differential urinary metabolites involved in the significant metabolism pathways between the case and control groups. **A** 5-Hydroxyindoleacetate. **B** L-Kynurenine. **C** 5-Hydroxy-L-Tryptophan. **D** 3-Hydroxyanthranilic acid. **E** 5-Methoxyindoleacetate. **F** Phenylpyruvic acid. **G** Hippuric acid. **H** The ROC curve of the above metabolites. a-g represented the metabolites 5-hydroxyindoleacetate, L-kynurenine, 5-hydroxy-L-tryptophan, 3-hydroxyanthranilic acid, 5-methoxyindoleacetate, phenylpyruvic acid and hippuric acid, respectively. The line in panel A-G represented the median concentration and the dots were the concentration values of the individuals. ROC curve, the receiver operator characteristic curve

Four key differential metabolic pathways were observed, including tryptophan metabolism, pentose phosphate pathway, phenylalanine metabolism and caffeine metabolism pathways (all *P* < 0.05) (Fig. 1D). A total of 10 metabolites contributed to the above four significant metabolism pathways by KEGG annotation. 5-hydroxyindoleacetate (5-HIAA), L-kynurenine (KYN), 5-hydroxy-L-tryptophan (5-HTP), 3-hydroxyanthranilic acid (3-HAA), and 5-methoxyindoleacetate (5-MIAA) were involved in the tryptophan metabolism pathway, with all these metabolites increasing except 5-MIAA in the case group compared to the control group (Fig. 2A–E, Additional file 9: Table S2). The ROC curve of the four increased metabolites showed good predictive abilities to distinguish the case group from the control group (AUC: 0.980 for 5-HIAA, 0.980 for KYN, 0.965 for 5-HTP, and 0.790 for 3-HAA, respectively) (Fig. 2H). The elevated phenylpyruvic acid and lowered hippuric acid were involved in phenylalanine metabolism pathway (AUC 0.922 and 0.740, Fig. 2F–H). Three metabolites including D-ribose, D-gluconic acid, and gluconolactone were involved in the pentose phosphate pathway and these metabolites all elevated in the urine samples of SFTS patients with the AUC of 0.882, 0.863, and 0.731, respectively (Additional file 1: Fig. S1A–C&F). Involved in the caffeine metabolism pathway, 3,7-Dimethyluric acid and theobromine lowered in SFTS patients with the AUC of 0.856 and 0.770, respectively (Additional file 1: Fig. S1D–F, Additional file 9: Table S2).

### Differential urinary metabolites between the fatal and survival groups

**Fig. 3:**
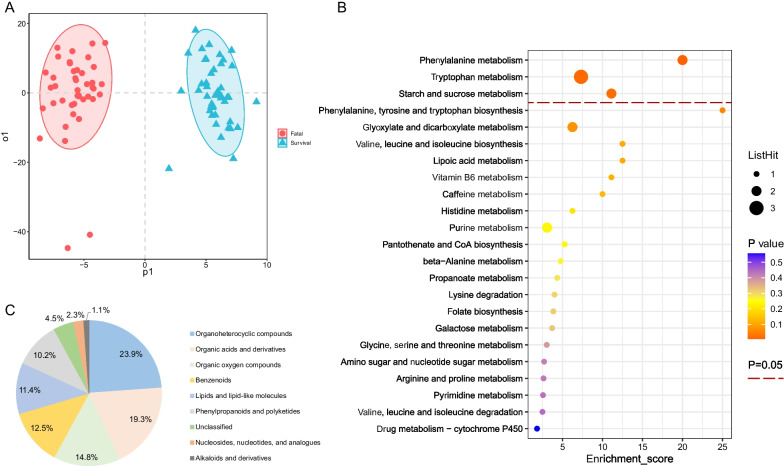
Differential urinary metabolites and metabolic pathways between the fatal and survival groups. **A** The OPLS-DA model of the two groups. **B** The super classes of significant differential urinary metabolites between the two groups. **C** The KEGG pathway mapping of significant differential urinary metabolites between the patients and healthy controls. KEGG, Kyoto Encyclopedia of Genes and Genomes database. OPLS-DA, orthogonal partial least square discriminate analysis

**Fig. 4:**
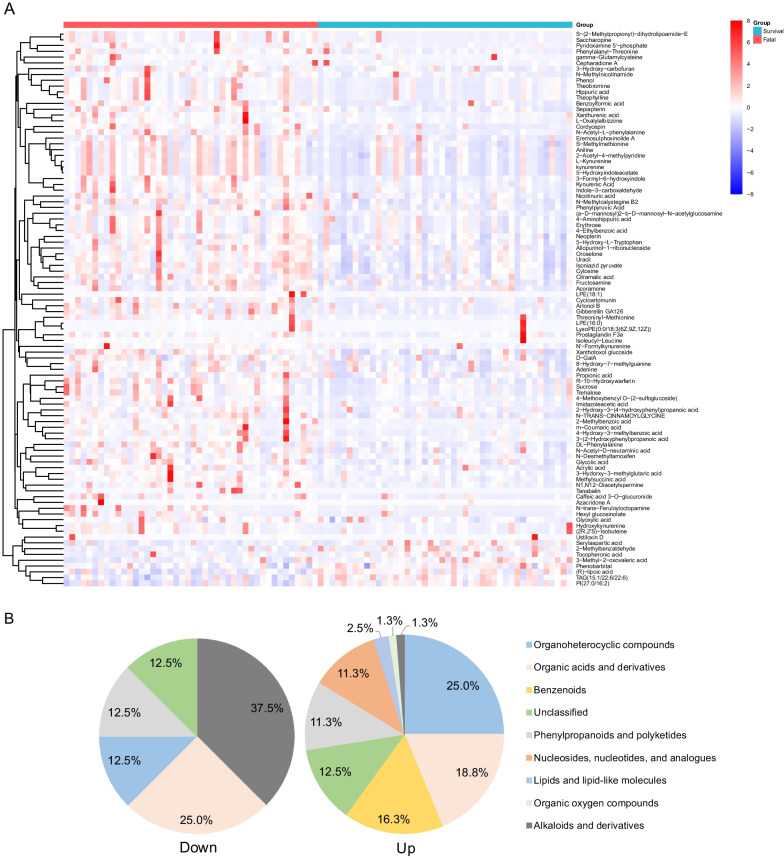
The differential urinary metabolites between the fatal and survival groups. **A** The heatmap of the differential urinary metabolites between the two groups. Color red represented higher concentration and color blue represented lower concentration. **B** The super classes of significantly up-regulated and down-regulated urinary metabolites of the fatal cases in contrast to the survival cases

Urinary metabolites were separated clearly between the fatal and survival groups supported by the OPLS-DA model (Fig. 3A). Totally 88 metabolites were selected as differential metabolites, which belonged to eight super classes. The top five were organoheterocyclic compounds (23.9%), organic acids and derivatives (19.3%), organic oxygen compounds (14.8%), benzenoids (12.5%), and lipids and lipid-like molecules (11.4%) (Fig. 3B). Among these differential metabolites, most of metabolites up regulated in the urinary metabolites (Fig. 4). There were 80 metabolites increased and 8 decreased among all the differential urinary metabolites. The top three super classes were organoheterocyclic compounds (25.0%), organic acids and derivatives (18.8%), benzenoids (16.3%) in the up-regulated ones. In the down-regulated ones, they belonged to alkaloids and derivatives (37.5%), organic acids and derivatives (25.0%), organoheterocyclic compounds (12.5%) and phenylpropanoids and polyketides (12.5%) (Fig. 4).

### Metabolic pathways between the fatal and survival groups

**Fig. 5:**
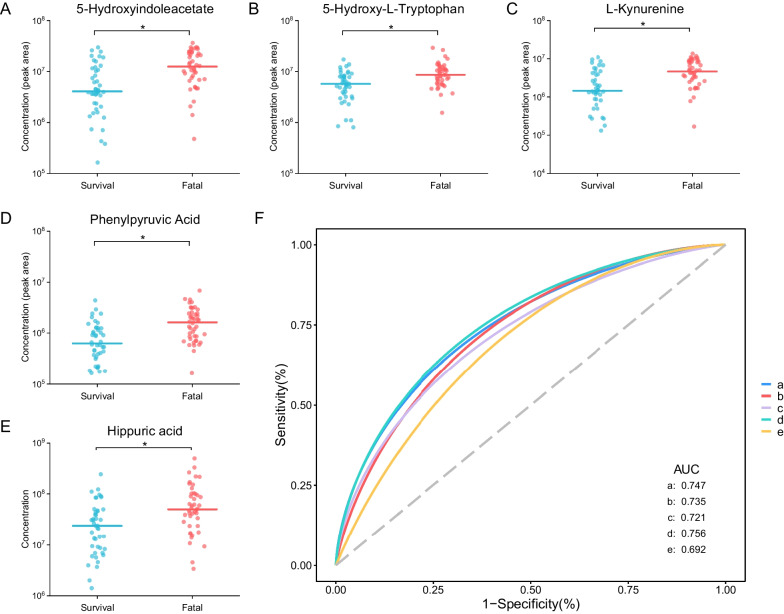
The concentration comparison of the differential urinary metabolites involved in the significant metabolism pathways between the fatal and survival groups. **A** 5-Hydroxyindoleacetate. **B** L-Kynurenine. **C** 5-Hydroxy-L-Tryptophan. **D** Phenylpyruvic acid. **E** Hippuric acid. **F** The ROC curve of the above metabolites. a-e represented the metabolites 5-hydroxyindoleacetate, L-kynurenine, 5-hydroxy-L-tryptophan, phenylpyruvic acid and hippuric acid, respectively. The line in panel A-E represented the median concentration and the dots were the concentration values of the individuals. ROC curve, the receiver operator characteristic curve

Three key differential metabolic pathways were observed, including phenylalanine metabolism, tryptophan metabolism, and starch and surcose metabolism pathways (all *P* < 0.05) (Fig. 3C). There were seven metabolites in all contributing to the above three significant metabolism pathways supported by KEGG annotation. 5-HIAA, KYN, and 5-HTP were involved in the tryptophan metabolism pathway, and all up-regulated in the fatal group compared to the survival group (Fig. 5A–C, Additional file 9: Table S3). The AUC of these metabolites were 0.747, 0.735, and 0.721, respectively (Fig. 5F). The same two urinary metabolites as that in the comparison of case and control groups were involved by phenylalanine metabolism pathway with phenylpyruvic acid and hippuric acid both elevating in the fatal group compared to the survival group (AUC: 0.756 and 0.692 respectively) (Fig. 5D–F, Additional file 9: Table S3). The starch and sucrose metabolism pathway hit two metabolites including trehalose and sucrose, and these metabolites all elevated in the fatal group with the AUC of 0.713 and 0.709, respectively (Additional file 2: Fig. S2A–C, Additional file 9: Table S3).

### Main differences in metabolic profile between the control and fatal groups

In the metabolic profile of the control and fatal groups, the two groups were separated obviously with the OPLS-DA model (Additional file 3: Fig. S3A). A total of 518 metabolites were differentiated from the control and fatal groups and classified into super classes mainly including lipids and lipid-like molecules (18.8%), organic acids and derivatives (16.6%) and organoheterocyclic compounds (13.9%) (Additional file 3: Fig. S3B). Among all the differential urinary metabolites, there were 431 up-regulated and 87 down-regulated in the fatal group compared to the control group (Additional file 3: Fig. S3C). Particularly, 5-HIAA, 5-HTP, KYN, 3-HAA and indoleacetic acid were involved in the tryptophan metabolism pathway, and all up-regulated except indoleacetic acid in the fatal group compared to the survival group (Additional file 4: Fig. S4A-E, Additional file 9: Table S4). The AUC of these metabolites were 0.996, 0.993, 0.983, 0.780 and 0.702, respectively (Additional file 4: Fig. S4F).

### Main differences in metabolic profile between the control and survival groups

The control and survival groups were separated obviously by metabolic profile using the OPLS-DA model (Additional file 5: Fig. S5A). A total of 488 metabolites were differentiated from the two groups and the top three super classes were the same as those between the control and case groups, and the control and fatal groups. They were lipids and lipid-like molecules (18.4%), organic acids and derivatives (16.2%) and organoheterocyclic compounds (13.3%) (Additional file 5: Fig. S5B). Among all the differential urinary metabolites, there were 431 up-regulated and 87 down-regulated in the fatal group compared to the control group (Additional file 5: Fig. S5C). 5-HIAA, 5-HTP, KYN, 3-HAA and 5-MIAA were involved in the tryptophan metabolism pathway, and all up-regulated except 5-MIAA in the fatal group compared to the survival group (Additional file 6: Fig.ure S6A-E, Additional file 9: Table S5). The AUC of these metabolites were 0.974, 0.946, 0.979, 0.823 and 0.680, respectively (Additional file 6: Fig. S6F).

### Serum glycine and amino acid supplement

**Fig. 6:**
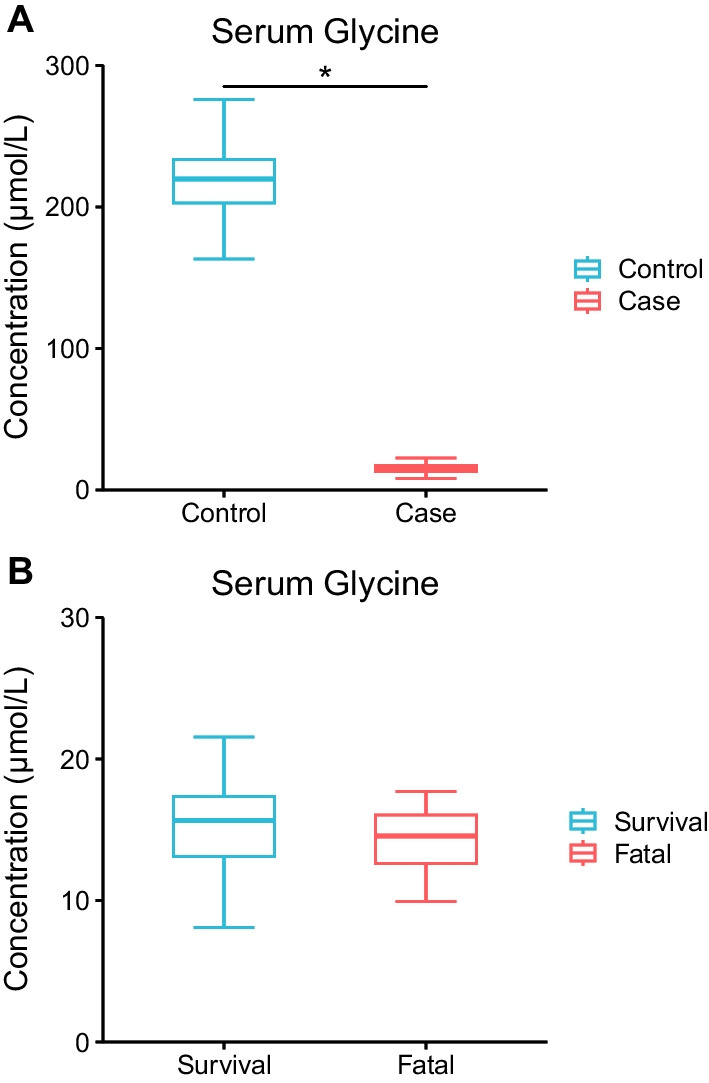
The comparison of serum glycine concentration between the case and control groups, and between the fatal and survival groups. **A** The serum glycine concentration in the case and control groups. **B** The serum glycine concentration in the fatal and survival groups. The lines in the middle and the top and bottom borders of the box represented the median and the upper and lower quartiles respectively. The top and bottom error bars represented the maximum and minimum respectively

The concentration of hippuric acid decreased in the urine of the case group compared to the control group, whereas it increased in the fatal group compared to the survival group. Considering the non-progressive decline of hippuric acid in urine among the control group, survival group and fatal group, we further analyzed the concentration of serum glycine and the amino acid supplement to figure out the potential factors rendering the phenomenon. Based on the previous results of targeting metabolomics for serum samples, the concentration of serum glycine greatly reduced in SFTS patients compared with health people (*P* < 0.001, Fig. 6A). There was no significant difference between the fatal and survival patients (*P* = 0.207, Fig. 6B). The median dose of compound amino acid injection, between the fatal patients and the survival patients showed no significant difference (250 *vs.* 250 mL/day, *P* = 0.751, Additional file 9: Table S6).

### Biochemical indexes

We evaluated a set of biochemical indexes related to hepatic function. The detection results were divided into before and after the urine sample collection and then be integrated by calculating the median for each patient in each period. The level of lactate dehydrogenase (LDH), aspartate transaminase (AST), alanine transaminase (ALT), total bilirubin (TBil), direct bilirubin (DBil), and indirect bilirubin (IBil) in serum were all no significantly different between the survival and fatal groups during the stage before the urine samples were obtained (all *P* > 0.05, Additional file 7: Fig. S7), indicating a comparable status of hepatic function between the two groups in this stage of SFTSV infection. After the urine sampled, however, the concentration of LDH, AST, ALT, and DBil in the fatal group elevated significantly compared to the survival group (all *P* < 0.05), while the concentration of IBil significantly declined (*P* = 0.001) and TBil showed no significant difference (Additional file 8: Fig. S8).

## Discussion

In this study, we depicted the urinary metabolic profile of SFTS patients and explored the metabolic alterations in fatal SFTS patients. The results indicated a series of differential urinary metabolites and the metabolism pathways they were involved in when comparing the control and case groups as well as the survival and fatal groups. Tryptophan metabolism and phenylalanine metabolism were the top one important metabolism pathway in differentiating the control and case groups, and the survival and fatal groups, respectively. Related metabolism pathways were also reported as significant in previous study [13]. The significant increase of differential metabolites in tryptophan metabolism, including 5-HIAA, KYN, 5-HTP and 3-HAA, and the increase of phenylpyruvic acid and decrease of hippuric acid in phenylalanine metabolism indicated the potential metabolic alterations in SFTSV infection. Among the metabolites above, 5-HIAA, KYN, 5-HTP, phenylpyruvic acid and hippuric acid might also act in the fatal progress of SFTS patients.

As consistent with previous findings [13], tryptophan metabolism was revealed as one of the important metabolism path ways in this study. Tryptophan, an essential amino acid in humans, is involved in several physiological processes including neuronal function, immunity, and gut homeostasis [16]. The majority of free tryptophan is degraded through the kynurenine pathway (KP) in mammals and generates a range of metabolites involved in inflammation, immune response, and excitatory neurotransmission [17]. In this study, we observed the increased concentration of urinary 5-HIAA, 5-HTP, and KYN, which are the metabolites in the pathway of tryptophan metabolism, both in patients infected by SFTSV compared with general population and fatal SFTS patients compared with survival patients. 5-HTP, 5-HIAA and KYN were all metabolic intermediates of tryptophan degradation, and 5-HIAA was the metabolite right downstream 5-HTP [18]. These results indicated that tryptophan probably contributed to the virus clearance and physical inflammation in SFTSV infection.

Typically, only small amounts of 5-HIAA are present in the urine of general population. Neutrophil transfer experiments established that GPR35 contributed to the efficiency with which these cells were recruited to sites of inflammation. Meanwhile, 5-HIAA was found as a ligand or agonist for GPR35 [18, 19], which indicated that the increased 5-HIAA in SFTS patients probably promoted the inflammation during the infection of SFTSV. The activity of KP is strictly regulated under healthy conditions but may be strongly influenced by inflammation [20]. Pathological changes of many neuroactive and immunomodulatory KP metabolites have been proved to be involved in immune system disruption and central nervous system alterations [21]. KYN was up-regulated in patients infected by SFTSV especially in fatal SFTS patients and possibly contributed to the abnormal biological processes, immune disorders and neural disruption during SFTSV infection as the increases of KYN could suppress and induce the apoptosis of Th1 and natural killer cells as well as down-regulate the CD8^+^ receptor expression, impairing their cytotoxic activity [22]. As to the decrease of 5-MIA, it might attribute to the reduction or inhibition of acetylserotonin O-methyltransferase, the enzyme catalyzing 5-HIAA degrading into 5-MIA and as well as the melatonin biosynthesis requires, and the suppression of melatonin has been reported to contribute to the initial cytokine storm in viral infections [23].

The inflammation could probably interfere with phenylalanine metabolism [24]. The urinary phenylpyruvic acid increased in SFTS patients especially those who deceased, which might be caused by the weakened the activity of phenylalanine hydroxylase (or cofactor). This could subsequently lead to the block of the main metabolic pathway of phenylalanine (metabolism to tyrosine) and finally result in the rise of phenylpyruvic acid in urine [24, 25]. The other differential metabolite in our study involved in the phenylalanine metabolism pathway, hippuric acid, has been reported to be a pivotal microbial–host co-metabolite mediating part of the beneficial metabolic improvements and turning out to be a biomarker of metabolic health by integrated analysis of metabolomics and metagenomics [26]. Hippuric acid is produced by the conjugation of glycine and benzoyl-coenzyme A (benzoyl-CoA) [27, 28]. Glycine availability is one of the most significant factors in determining the rate of hippuric acid production, as well as the depletion of benzoyl-CoA has been implicated as a rate-limiting factor [28]. We also observed a huge reduction of the serum glycine level among SFTS patients compared to healthy people according to previous targeting metabolomics results. Glycine is involved in the metabolism of proteins, nucleotide, porphyrin and bile salts, as well as a neurotransmitter in the central nervous system, and it also has broad-spectrum anti-inflammatory, cell protection and immunomodulatory effects [29]. Thus, the decrease of hippuric acid in the urine of SFTS patients, probably a result of the reduction of glycine in the serum during SFTSV infection, might transmit a poorer health status of gut microbiota and microenvironment of infected patients and indicated that glycine might also play an important role in the invasion of this virus [26]. Inversely, the concentration of hippuric acid elevated in the fatal cases compared with the survival cases, whereas the serum glycine level was low in fatal cases with previous detection supported and no significant difference was observed of the urinary glycine level or the supplement of amino acids between the two groups, declaring a rise of benzoyl-CoA in fatal cases, which might attributed to the augmented exogenous benzoic acid, which was harmful to human health and detoxified in the liver, as well as to the alteration of the interaction between the gut microbiota and the aromatic compounds based on that benzoyl-CoA is the intermediate in the degradation of aromatic compounds by cells or bacteria including gut microbiota [27, 30]. For a further understanding of the relationship, we evaluated the biochemical indexes linked with the hepatic function in the survival and fatal groups and found that the status of hepatic function between the two groups were comparable initially but the fatal cases turned to have a more abnormal hepatic status compared to the survival cases afterwards, suggesting the metabolic load might increase more for the fatal cases, which supported the assumption above that their liver worked harder to metabolize harmful chemical substances like benzoic acid resulting in an increase of downstream metabolites like hippuric acid. However, the relationships remained unclear and needs further evidence.

Based on the results that urinary metabolites of SFTS patients, tryptophan metabolism and phenylalanine metabolism might be involved in the mechanism of SFTSV invasion and lethal mechanism. As the mechanism of how the key metabolism pathways function in the pathogenesis of SFTSV infection has not been clear, we presumed the assumptions as follows: First, the alteration of the metabolites involved in the tryptophan metabolism and phenylalanine metabolism might act as a helper for the overproduction of cytokines which could trigger a dangerous situation known as a cytokine storm and then lead to a more severe or even fatal outcome [31]. Second, previous studies have revealed that B cell is probably one of the targets in SFTSV infection [32, 33]. Meanwhile, tryptophan and kynurenine may promote the proliferation of B cells, inhibits the somatic hypermutation and class switch recombination of B cells, and inhibits the differentiation of plasma blast and plasma cells [34]. Thus, tryptophan might influence the attack process of B cells in the SFTSV invasion. Third, studies have shown that endothelial injury is an important pathogenesis of SFTS, but the molecular mechanism is unknown [35, 36]. Tryptophan metabolism has been reported as a key pathway to regulate vascular endothelial function [37]. Accordingly, we speculate that abnormal tryptophan metabolism may be related to vascular endothelial injury in SFTS. However, these speculations have not been verified. There was also a possibility that these metabolism pathways might be part of the manifestation during the process of the resistance against SFTSV infection. The specific causal relationship and the role of tryptophan metabolism and phenylalanine metabolism in the pathogenesis of SFTSV infection needs further research to determine.

In addition, not specific to SFTSV infection, the variation of tryptophan metabolism and phenylalanine metabolism were also observed as linked to the biological process of other viral or bacterial infections such as HIV infection, SARS-CoV-2 infection, mycobacterium tuberculosis (Mtb) infection and so on [38–47]. The tryptophan metabolism is intimately associated with the regulation of diverse physiological processes. Accumulating evidence focusing on the pathophysiological changes caused by SARS-CoV-2 infection indicated a significant role for variations in tryptophan metabolism [39]. Serum phenylalanine was also observed increased in SARS-CoV-2 infected patients and various metabolomics studies have reported phenylalanine level probably provided a major contribution to the variability of severity in COVID-19 patients [40, 41]. There was probably a progressive and systemic induction of tryptophan oxidation partly driven by viral proteins in HIV infection, which might be a critical pathogenic part that over activates a normal host-immune strategy in HIV patients [38]. Tryptophan metabolism was highly regulated to produce KYN in tuberculosis (TB) disease [42]. Serum indoleamine 2,3-dioxygenase (IDO) activity was higher in active TB subjects than latently Mtb-infected individuals, which significantly declined in those after standard TB treatment, indicating that IDO might be a potential target in TB disease, and IDO activity and metabolite changes in tryptophan metabolism could be a predictor in the onset of TB disease [43, 45]. A comparative urine metabolome study showed altered metabolism of phenylalanine in TB patients [47], and change of phenylalanine level in serum samples was also observed in previous studies with TB patients or Mtb-infected animal models [45, 46].

Some limitations of this study need to be stated. First, the consideration on comorbidities and drug therapy was not sufficient which might cause bias on the metabolic analysis and pathway annotation. However, we have excluded the patients with comorbidities like diabetes and hepatitis that were more likely to alter the metabolic progress and matched the SFTS patients by receiving favipiravir therapy or not, both of which probably weakened the bias. Second, only urine samples were detected for metabolic profiling and we haven’t explored the correlation or difference of the metabolic profiling between urine and serum samples. Another, we haven’t further verified the cellular or molecular mechanism that the significant metabolism pathways and differential urinary metabolites involved in the pathogenesis of SFTS. Further research is needed to dig out the in-depth mechanism to support and elucidate the pathogenesis of SFTSV infection.

Despite these, our work depicted the urinary metabolic profile of SFTS patients and explored the metabolic alterations in fatal SFTS patients, which shed a new light on the potential pathogenesis of SFTSV invasion. This study mainly revealed tryptophan metabolism and phenylalanine metabolism might be involved in the pathogenesis of SFTSV infection. Also, it provided new underlying targets such as tryptophan and glycine for the diagnosis and treatment of SFTS. Functional verification and randomized controlled trials are indispensable in further research to clarify the pathogenic mechanism and the effects of such targets.

### Supplementary Information

**Additional file 1: Fig. S1.** The concentration comparison of the differential urinary metabolites involved in the other significant metabolism pathways between the case and control groups. A, D-Ribose. B, D-Gluconic Acid. C, Gluconolactone. D, 3,7-Dimethyluric acid. E, Theobromine. F, The ROC curve of the above metabolites. a-e represented the metabolites D-ribose, D-gluconic acid, gluconolactone, 3,7-dimethyluric acid, and theobromine, respectively. The line in panel A-E represented the median concentration and the dots were the concentration values of the individuals. ROC curve, the receiver operator characteristic curve

**Additional file 2: Fig. S2.** The concentration comparison of the differential urinary metabolites involved in the other significant metabolism pathways between the fatal and survival groups. A, Trehalose. B, Sucrose. C, The ROC curve of the above metabolites. a and b represented the same urinary metabolites as above. The line in A and B represented the median concentration and the dots were the concentration values of the individuals. ROC curve, the receiver operator characteristic curve

**Additional file 3: Fig. S3.** Differential urinary metabolites and metabolic pathways between the control and fatal groups. A, The OPLS-DA model of the two groups. B, The super classes of significant differential urinary metabolites between the two groups. C, The volcano plot and super classes of significantly up-regulated and down-regulated urinary metabolites of the fatal group in contrast to the control group. D, The KEGG pathway mapping of significant differential urinary metabolites between the two groups. KEGG, Kyoto Encyclopedia of Genes and Genomes database. OPLS-DA, orthogonal partial least square discriminate analysis

**Additional file 4: Fig. S4.** The concentration comparison of the differential urinary metabolites involved in the significant metabolism pathways between the control and fatal groups. A, 5-Hydroxyindoleacetate. B, 5-Hydroxy-L-Tryptophan. C, L-Kynurenine. D, 3-Hydroxyanthranilic acid. E, Indoleacetic acid. F, The ROC curve of the above metabolites. a-e represented the metabolites 5-hydroxyindoleacetate, 5-hydroxy-L-tryptophan, L-kynurenine, 3-Hydroxyanthranilic acid and indoleacetic acid, respectively. The line in panel A-E represented the median concentration and the dots were the concentration values of the individuals. ROC curve, the receiver operator characteristic curve

**Additional file 5: Fig. S5.** Differential urinary metabolites and metabolic pathways between the control and survival groups. A, The OPLS-DA model of the two groups. B, The super classes of significant differential urinary metabolites between the two groups. C, The volcano plot and super classes of significantly up-regulated and down-regulated urinary metabolites of the fatal group in contrast to the control group. D, The KEGG pathway mapping of significant differential urinary metabolites between the two groups. KEGG, Kyoto Encyclopedia of Genes and Genomes database. OPLS-DA, orthogonal partial least square discriminate analysis

**Additional file 6: Fig. S6.** The concentration comparison of the differential urinary metabolites involved in the significant metabolism pathways between the control and survival groups. A, 5-Hydroxyindoleacetate. B, 5-Hydroxy-L-Tryptophan. C, L-Kynurenine. D, 3-Hydroxyanthranilic acid. E, 5-Methoxyindoleacetate. F, The ROC curve of the above metabolites. a-e represented the metabolites 5-hydroxyindoleacetate, 5-hydroxy-L-tryptophan, L-kynurenine, 3-Hydroxyanthranilic acid and 5-methoxyindoleacetate, respectively. The line in panel A-E represented the median concentration and the dots were the concentration values of the individuals. ROC curve, the receiver operator characteristic curve

**Additional file 7: Fig. S7.** The comparison of biochemical index level between the case and control groups. A, LDH, lactate dehydrogenase. B, AST, aspartate transaminase. C, ALT, alanine transaminase. D, TBil, total bilirubin. E, DBil, direct bilirubin. F, IBil, indirect bilirubin. The lines in the middle and the top and bottom borders of the box represented the median and the upper and lower quartiles respectively. The top and bottom error bars represented the maximum and minimum respectively

**Additional file 8: Fig. S8.** The comparison of biochemical index level between the fatal and survival groups. A, LDH, lactate dehydrogenase. B, AST, aspartate transaminase. C, ALT, alanine transaminase. D, TBil, total bilirubin. E, DBil, direct bilirubin. F, IBil, indirect bilirubin. The lines in the middle and the top and bottom borders of the box represented the median and the upper and lower quartiles respectively. The top and bottom error bars represented the maximum and minimum respectively

**Additional file 9: Supplementary methods and Table S1-S6**. The detailed methods for quasi-targeted metabolomics of serum samples were described at the beginning, followed by Table S1-S6 which showed the list of hematologic indexes obtained in the laboratory tests, the concentration of differential metabolites between the control and case groups, the concentration of differential metabolites between the survival and fatal groups, the concentration of differential metabolites between the control and fatal groups, the concentration of differential metabolites between the control and survival groups, and the usage of compound amino acid injection in the survival and fatal groups.

## Data Availability

The datasets used and/or analyzed during the current study are available from the corresponding author on reasonable request.
