# Supplementary figures and images for: Metabolic alterations in urine among the patients with severe fever with thrombocytopenia syndrome
Source: Virol J. 2024 Jan 8;21:11. doi: 10.1186/s12985-024-02285-2 (PMC10775654; doi:10.1186/s12985-024-02285-2)

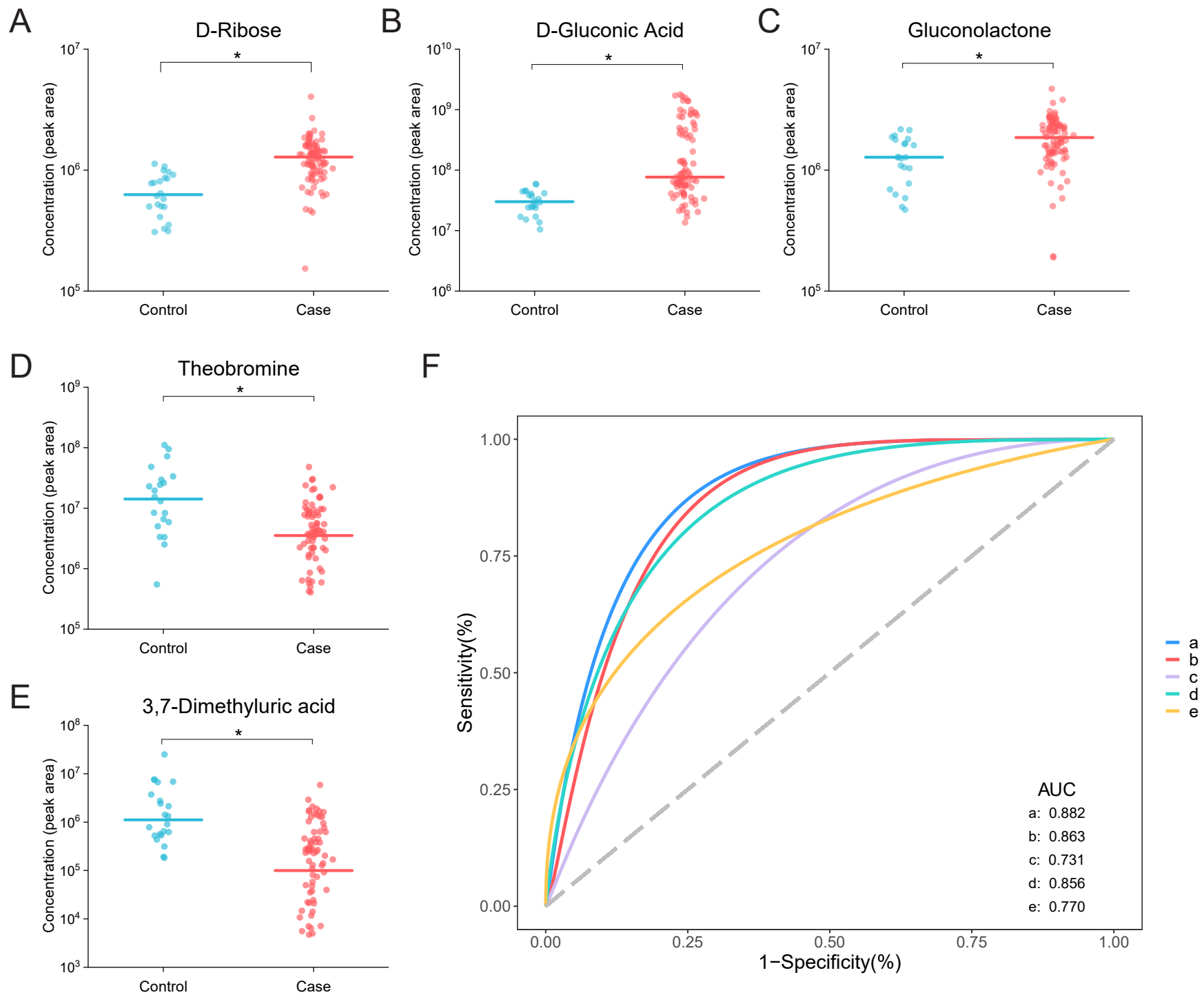

Supplement: Supplementary file 1 — Additional file 1: Fig. S1. The concentration comparison of the differential urinary metabolites involved in the other significant metabolism pathways between the case and control groups. A, D-Ribose. B, D-Gluconic Acid. C, Gluconolactone. D, 3,7-Dimethyluric acid. E, Theobromine. F, The ROC curve of the above metabolites. a-e represented the metabolites D-ribose, D-gluconic acid, gluconolactone, 3,7-dimethyluric acid, and theobromine, respectively. The line in panel A-E represented the median concentration and the dots were the concentration values of the individuals. ROC curve, the receiver operator characteristic curve [file 12985_2024_2285_MOESM1_ESM.pdf]

**A**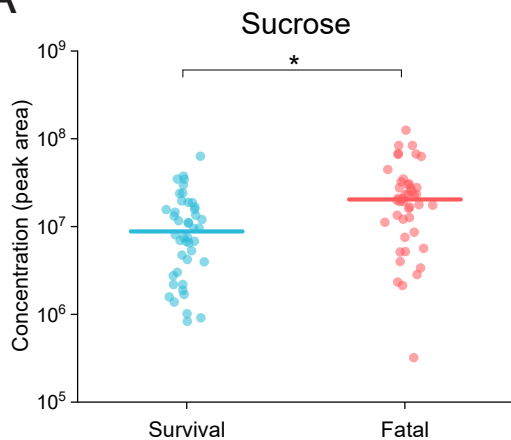**B**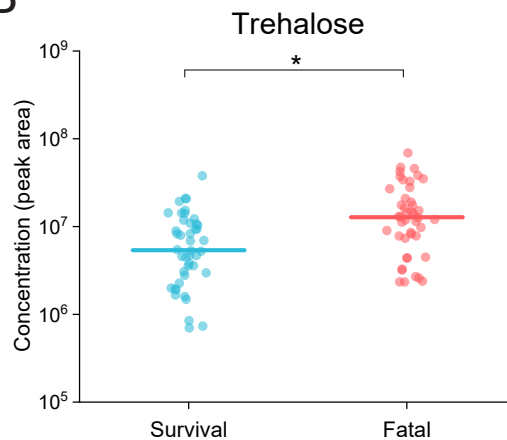**C**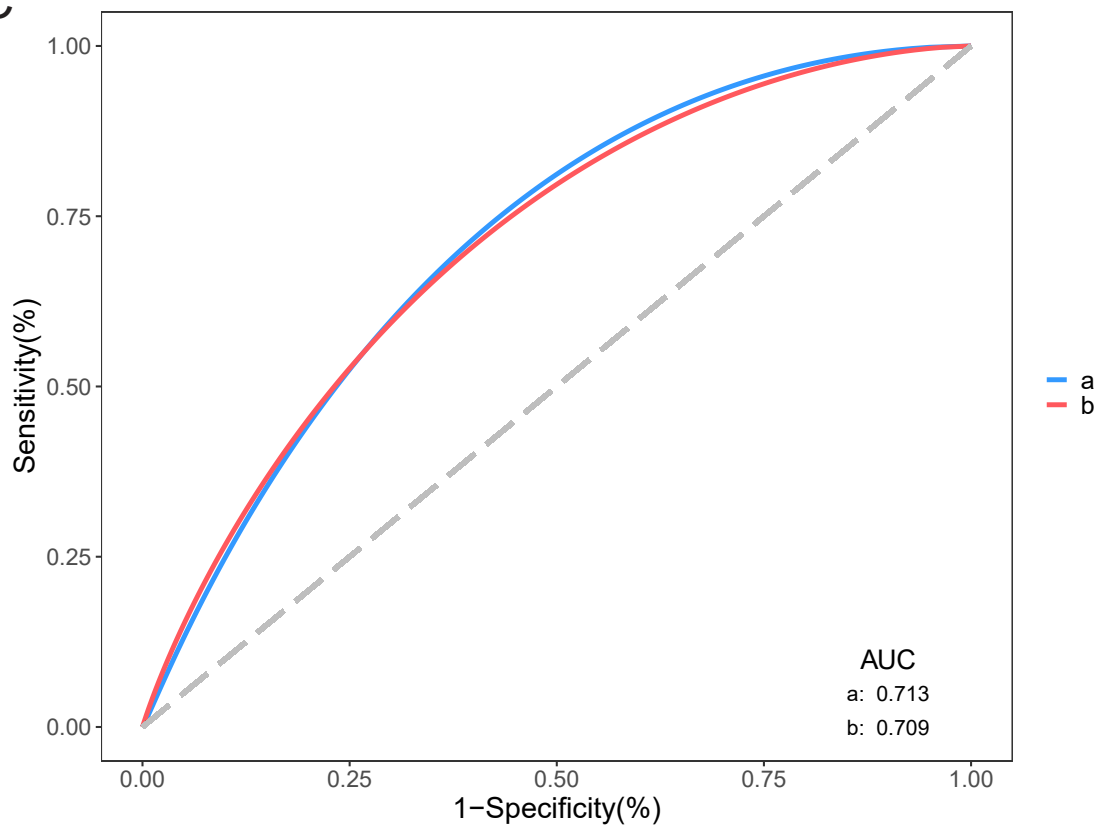

Supplement: Supplementary file 2 — Additional file 2: Fig. S2. The concentration comparison of the differential urinary metabolites involved in the other significant metabolism pathways between the fatal and survival groups. A, Trehalose. B, Sucrose. C, The ROC curve of the above metabolites. a and b represented the same urinary metabolites as above. The line in A and B represented the median concentration and the dots were the concentration values of the individuals. ROC curve, the receiver operator characteristic curve [file 12985_2024_2285_MOESM2_ESM.pdf]

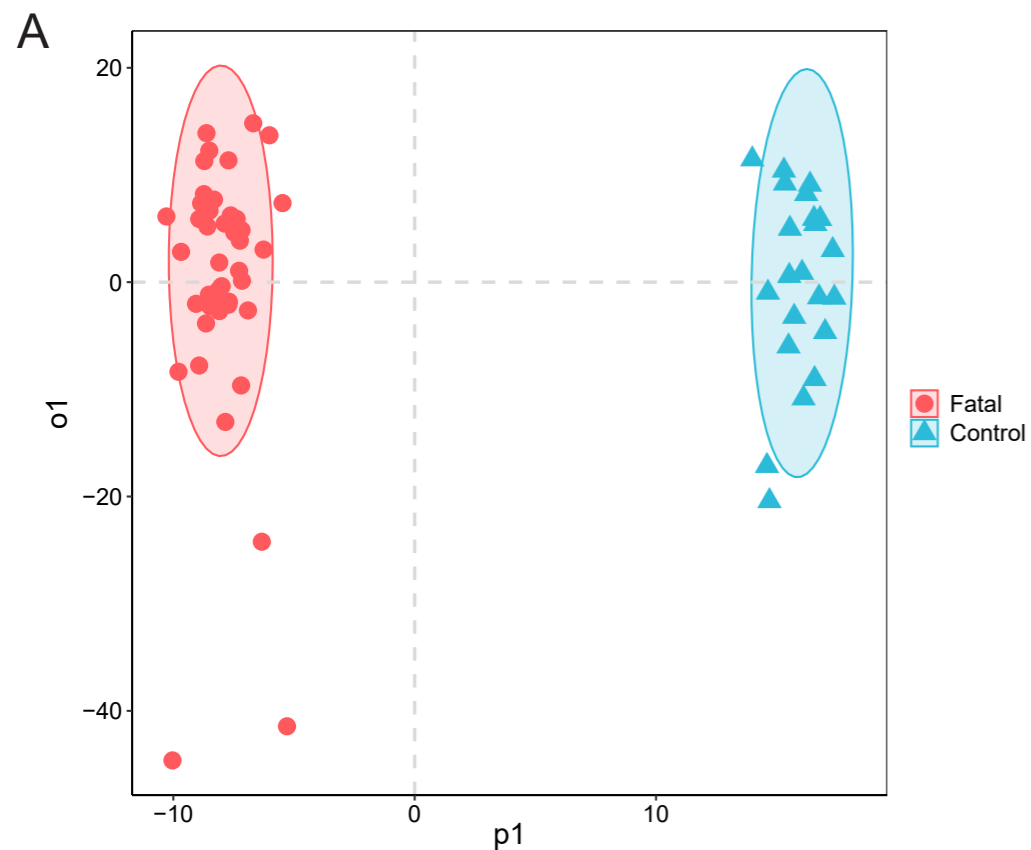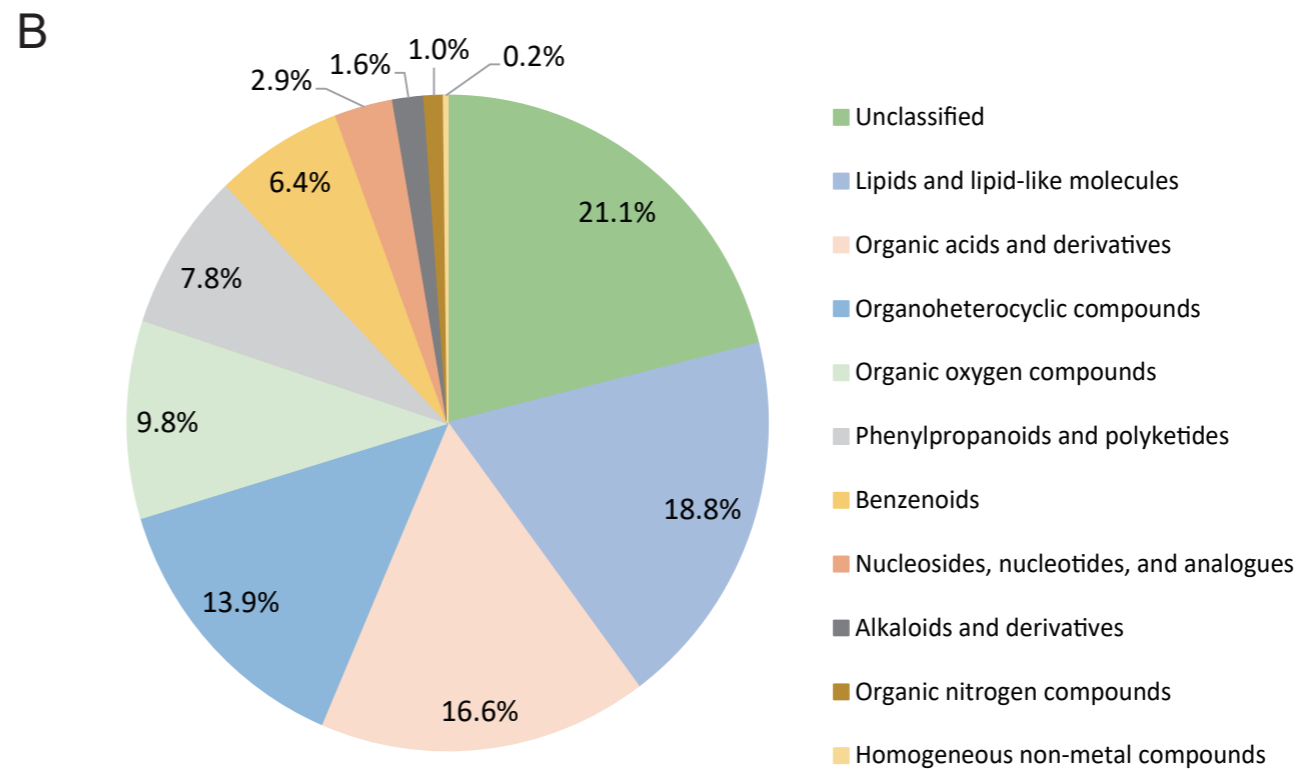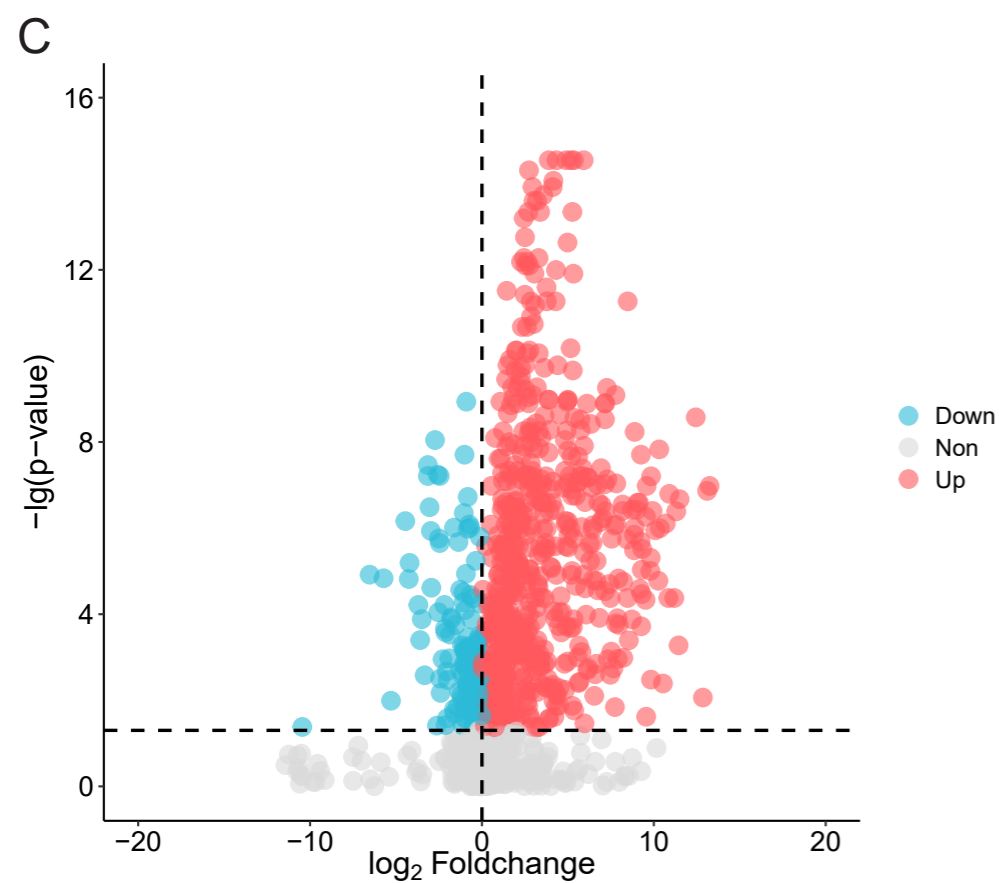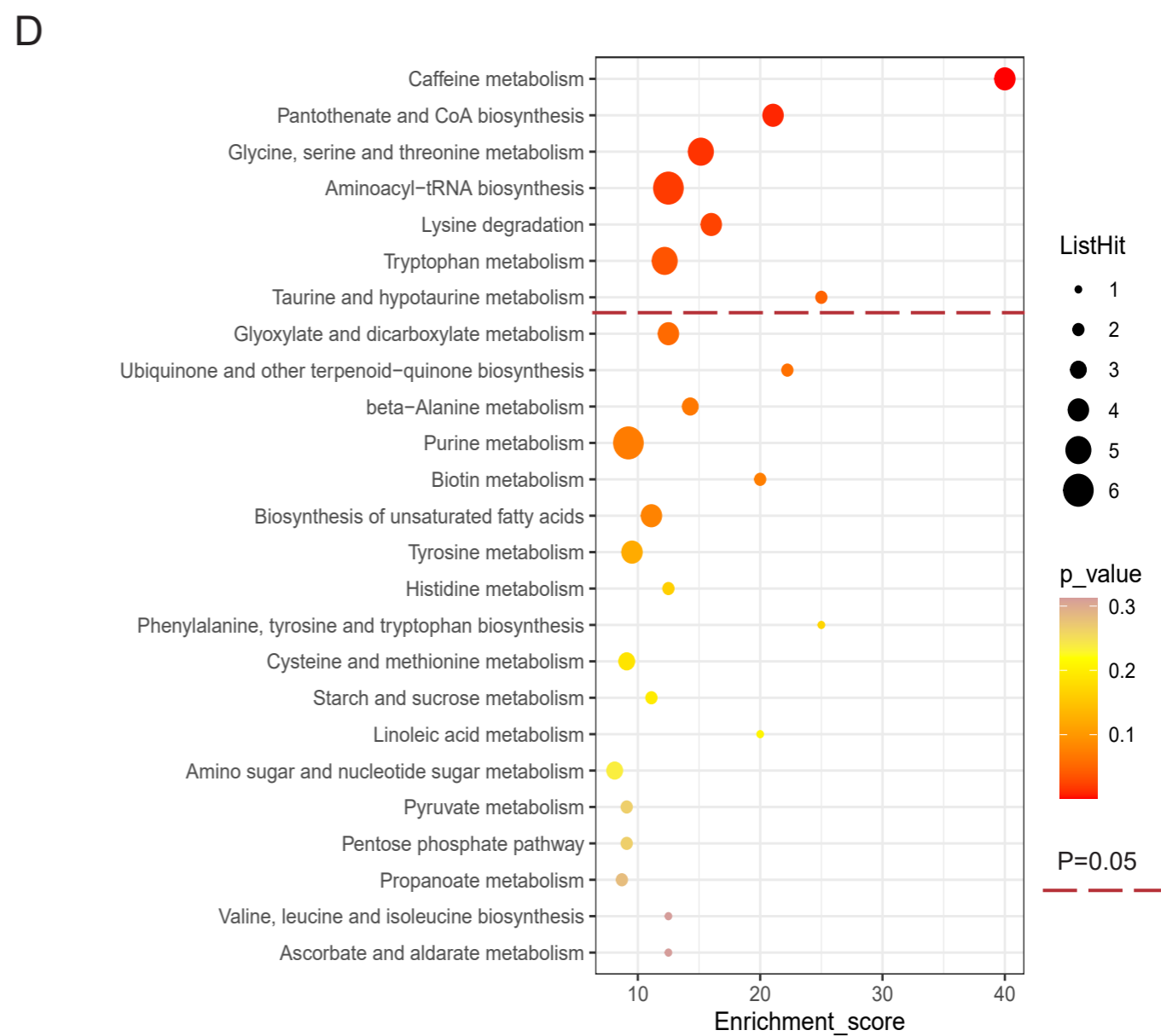

Supplement: Supplementary file 3 — Additional file 3: Fig. S3. Differential urinary metabolites and metabolic pathways between the control and fatal groups. A, The OPLS-DA model of the two groups. B, The super classes of significant differential urinary metabolites between the two groups. C, The volcano plot and super classes of significantly up-regulated and down-regulated urinary metabolites of the fatal group in contrast to the control group. D, The KEGG pathway mapping of significant differential urinary metabolites between the two groups. KEGG, Kyoto Encyclopedia of Genes and Genomes database. OPLS-DA, orthogonal partial least square discriminate analysis [file 12985_2024_2285_MOESM3_ESM.pdf]

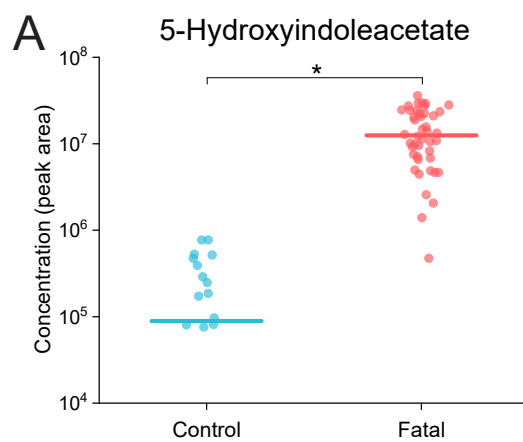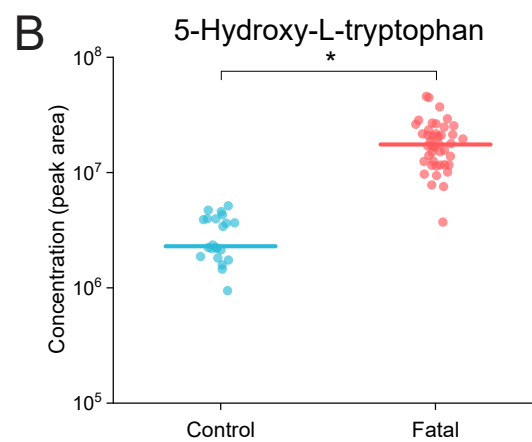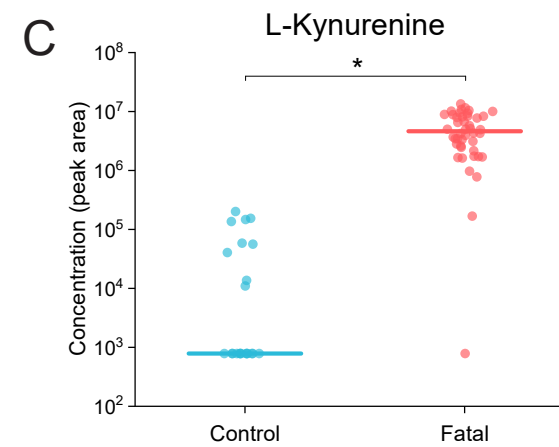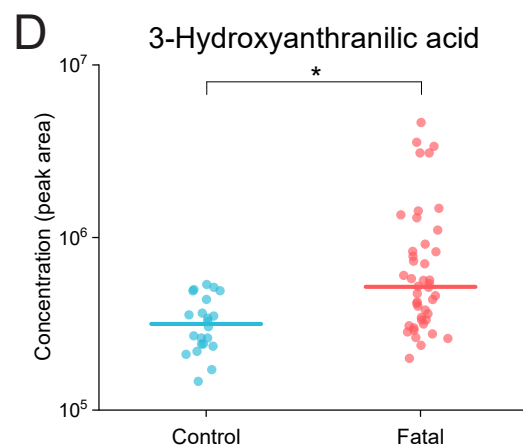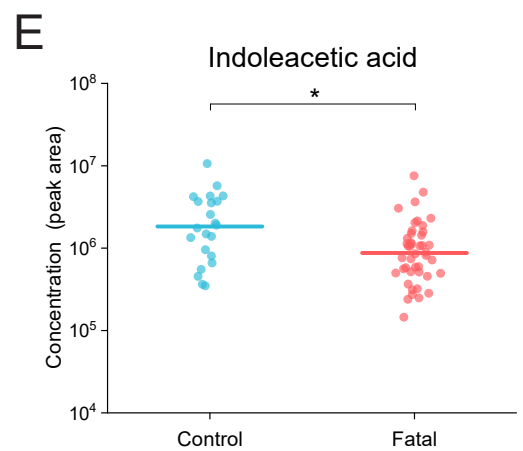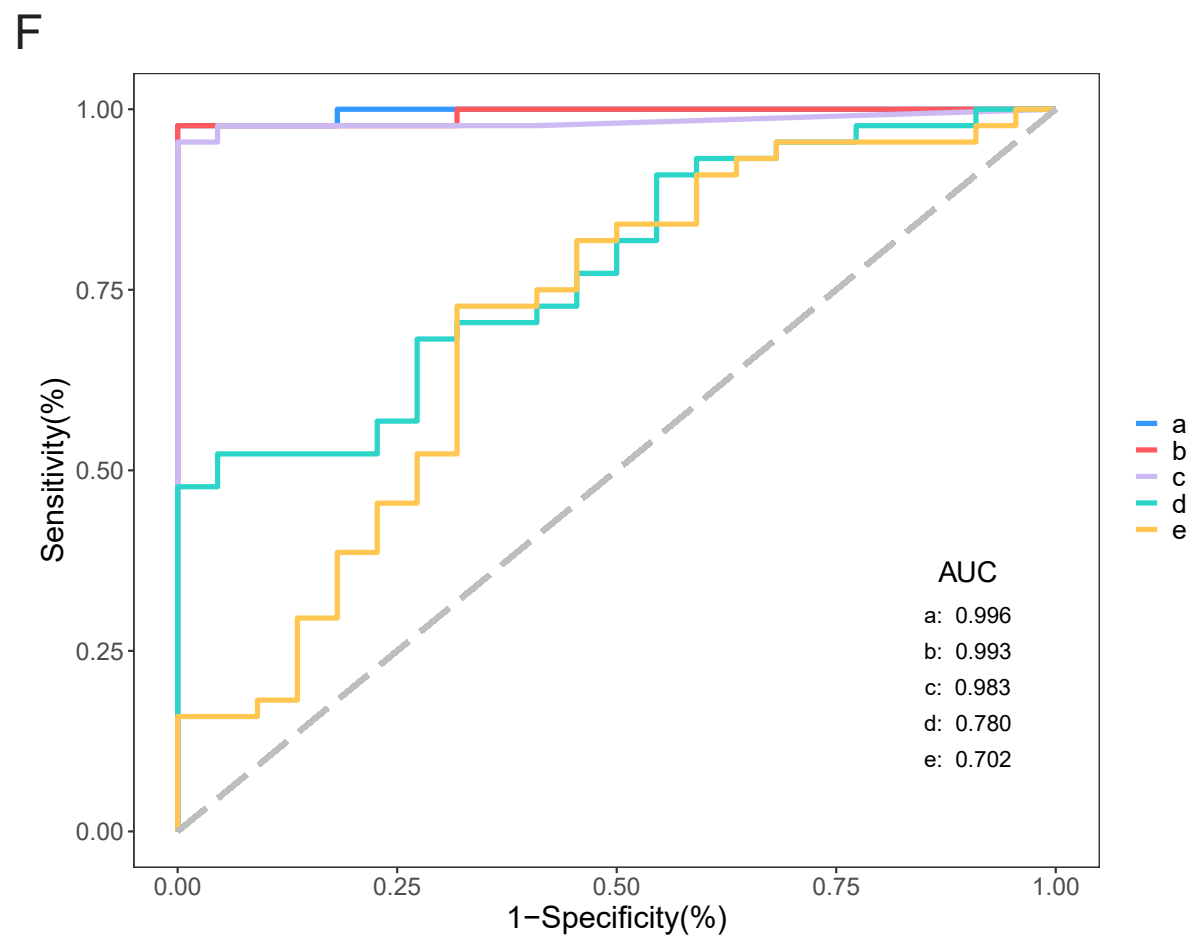

Supplement: Supplementary file 4 — Additional file 4: Fig. S4. The concentration comparison of the differential urinary metabolites involved in the significant metabolism pathways between the control and fatal groups. A, 5-Hydroxyindoleacetate. B, 5-Hydroxy-L-Tryptophan. C, L-Kynurenine. D, 3-Hydroxyanthranilic acid. E, Indoleacetic acid. F, The ROC curve of the above metabolites. a-e represented the metabolites 5-hydroxyindoleacetate, 5-hydroxy-L-tryptophan, L-kynurenine, 3-Hydroxyanthranilic acid and indoleacetic acid, respectively. The line in panel A-E represented the median concentration and the dots were the concentration values of the individuals. ROC curve, the receiver operator characteristic curve [file 12985_2024_2285_MOESM4_ESM.pdf]

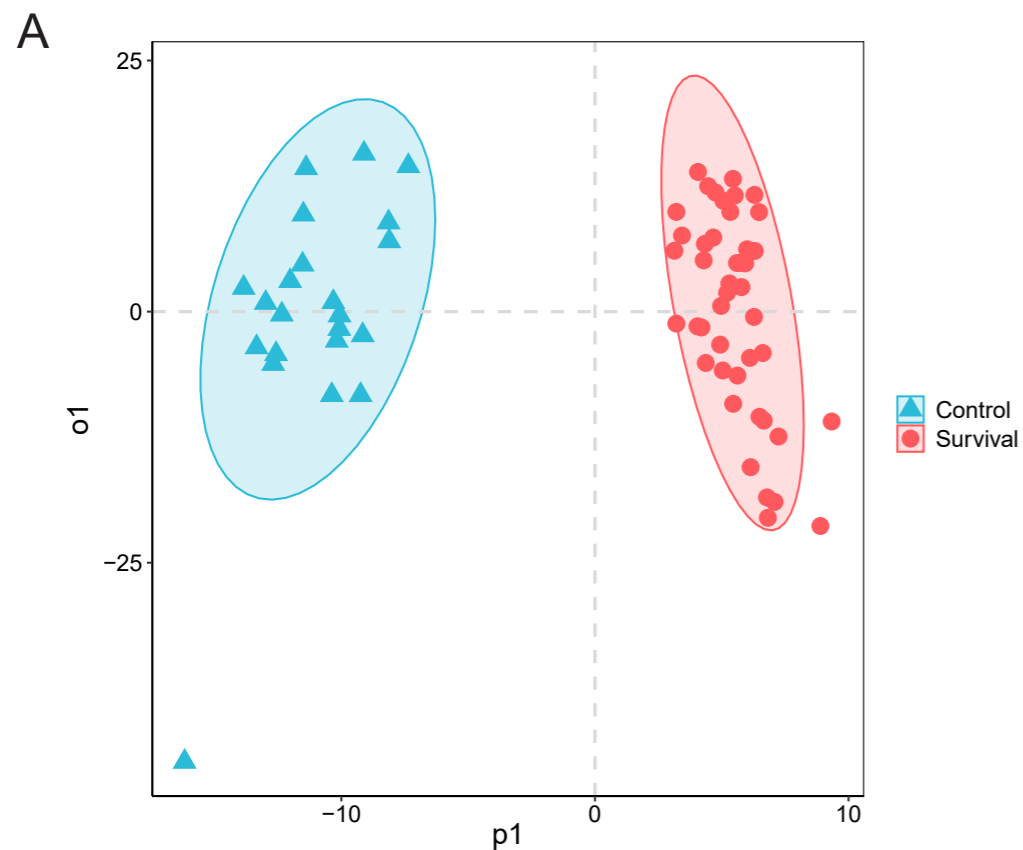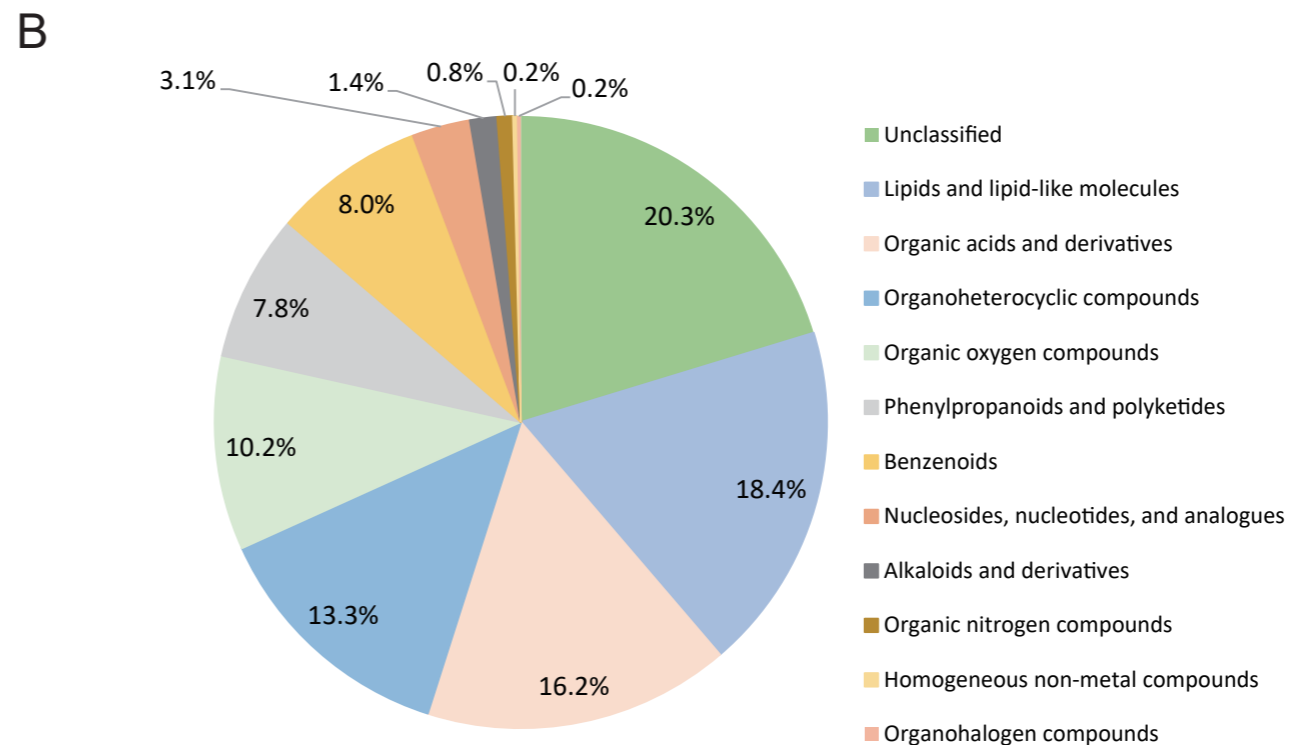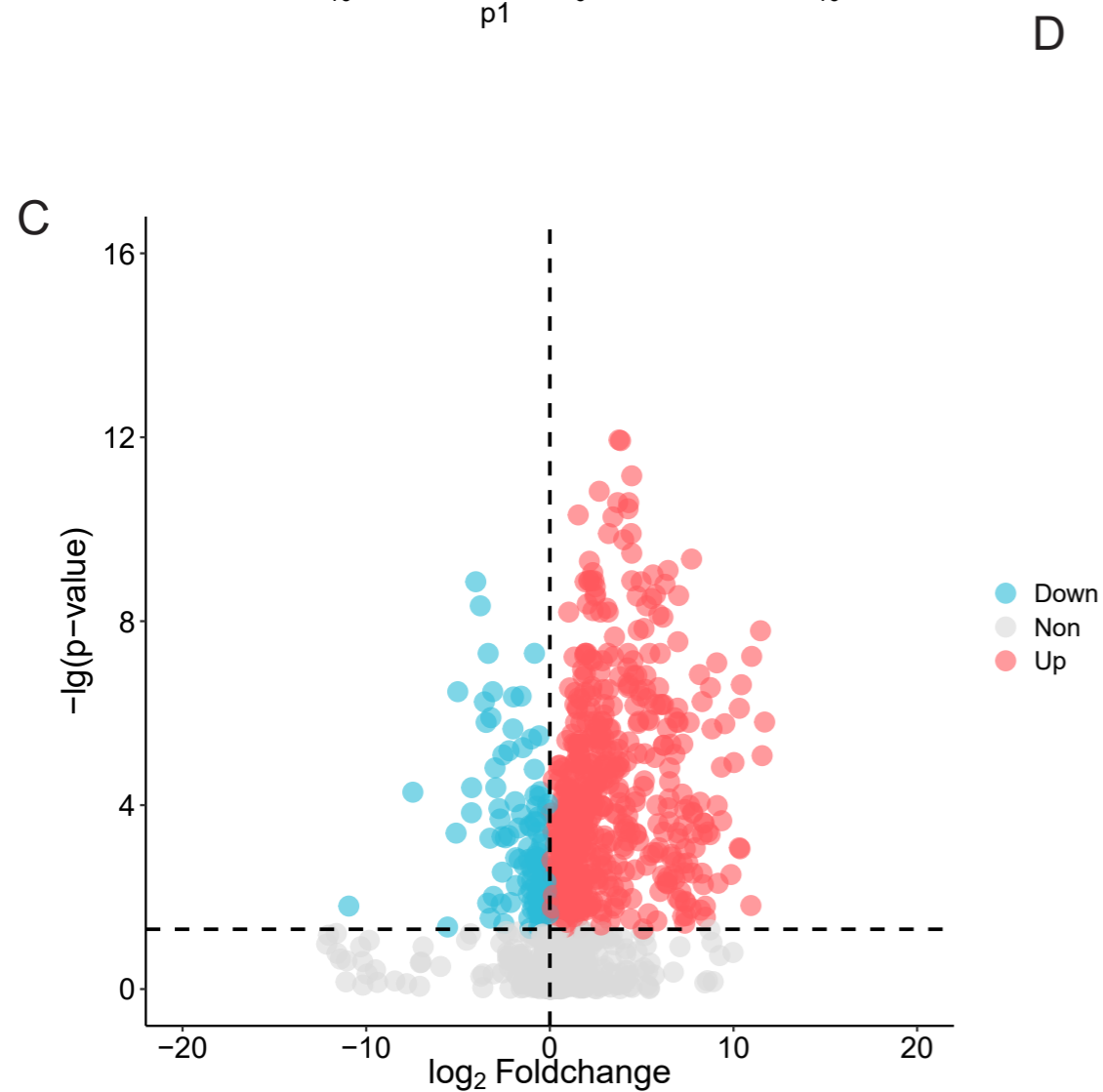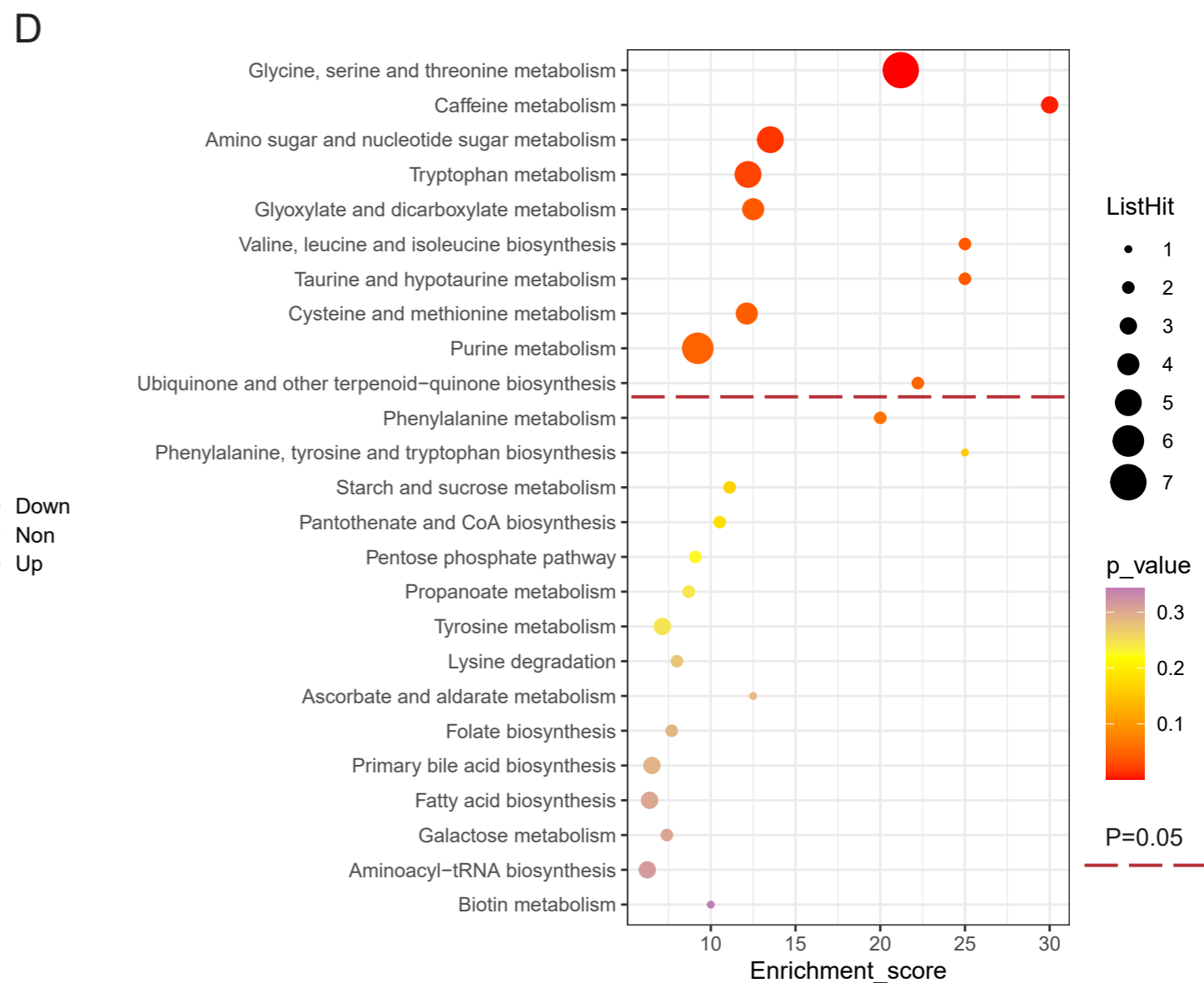

Supplement: Supplementary file 5 — Additional file 5: Fig. S5. Differential urinary metabolites and metabolic pathways between the control and survival groups. A, The OPLS-DA model of the two groups. B, The super classes of significant differential urinary metabolites between the two groups. C, The volcano plot and super classes of significantly up-regulated and down-regulated urinary metabolites of the fatal group in contrast to the control group. D, The KEGG pathway mapping of significant differential urinary metabolites between the two groups. KEGG, Kyoto Encyclopedia of Genes and Genomes database. OPLS-DA, orthogonal partial least square discriminate analysis [file 12985_2024_2285_MOESM5_ESM.pdf]

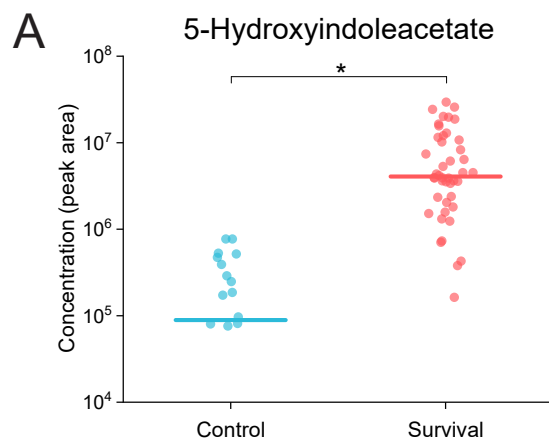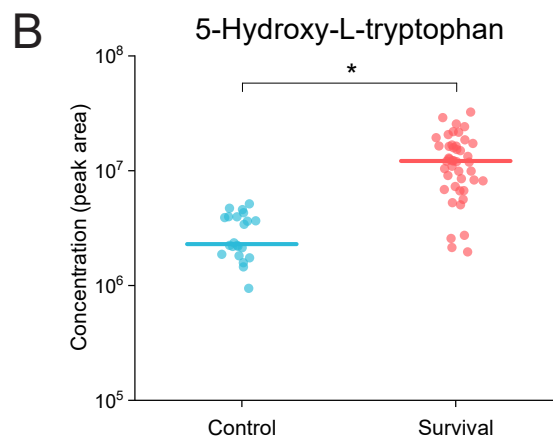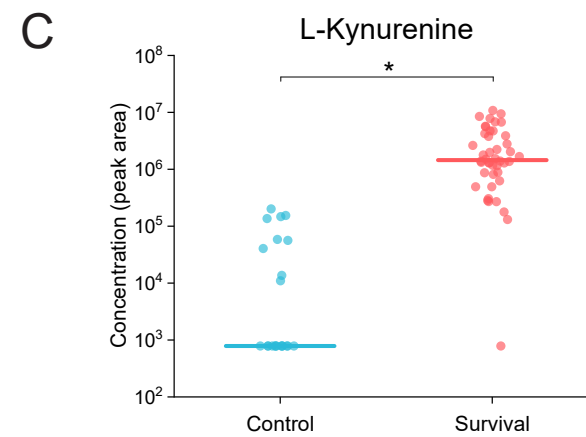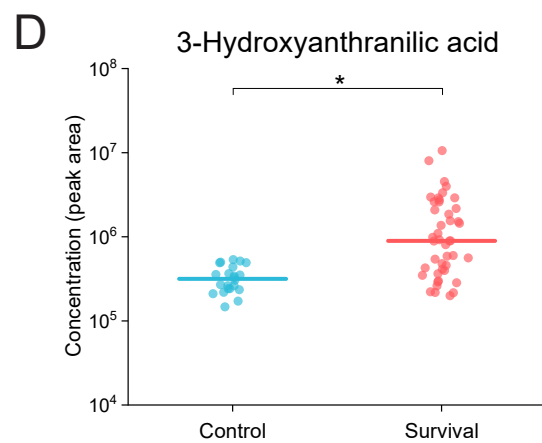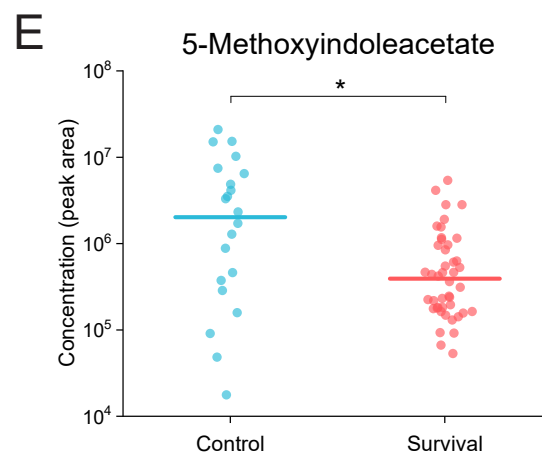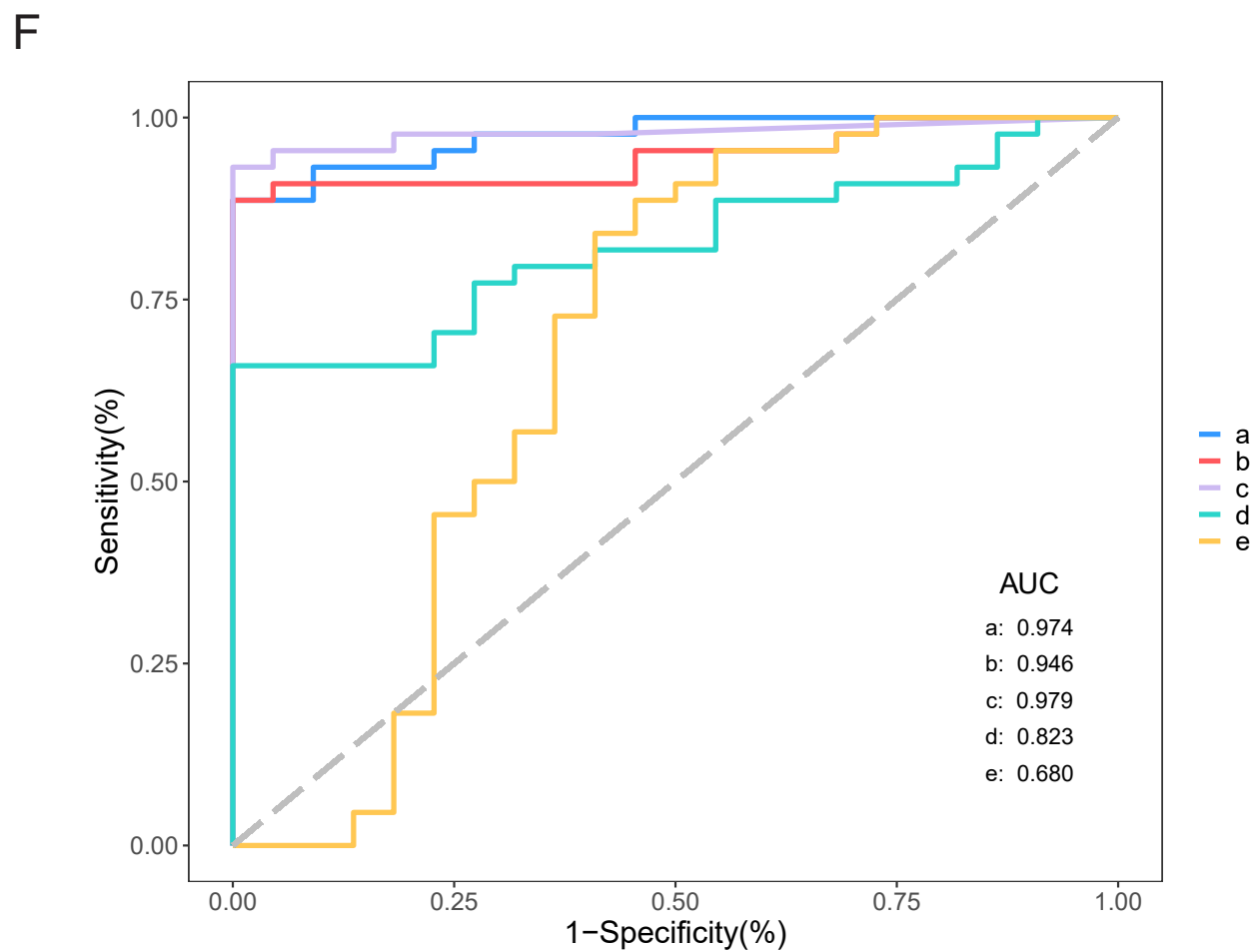

Supplement: Supplementary file 6 — Additional file 6: Fig. S6. The concentration comparison of the differential urinary metabolites involved in the significant metabolism pathways between the control and survival groups. A, 5-Hydroxyindoleacetate. B, 5-Hydroxy-L-Tryptophan. C, L-Kynurenine. D, 3-Hydroxyanthranilic acid. E, 5-Methoxyindoleacetate. F, The ROC curve of the above metabolites. a-e represented the metabolites 5-hydroxyindoleacetate, 5-hydroxy-L-tryptophan, L-kynurenine, 3-Hydroxyanthranilic acid and 5-methoxyindoleacetate, respectively. The line in panel A-E represented the median concentration and the dots were the concentration values of the individuals. ROC curve, the receiver operator characteristic curve [file 12985_2024_2285_MOESM6_ESM.pdf]

Survival Fatal

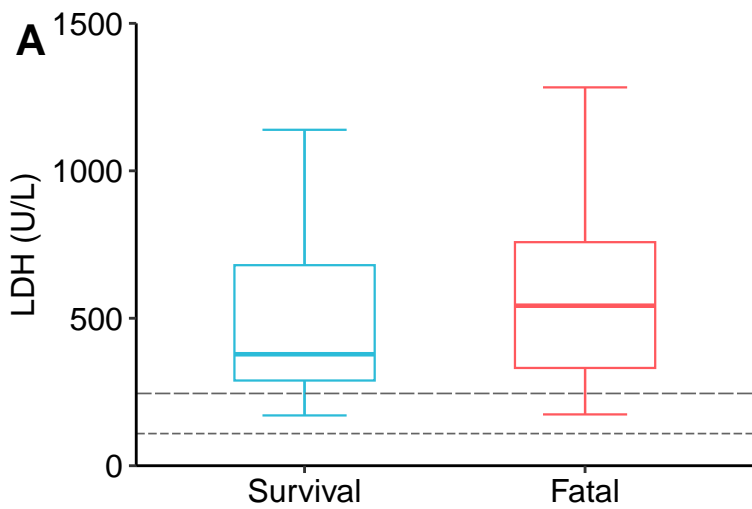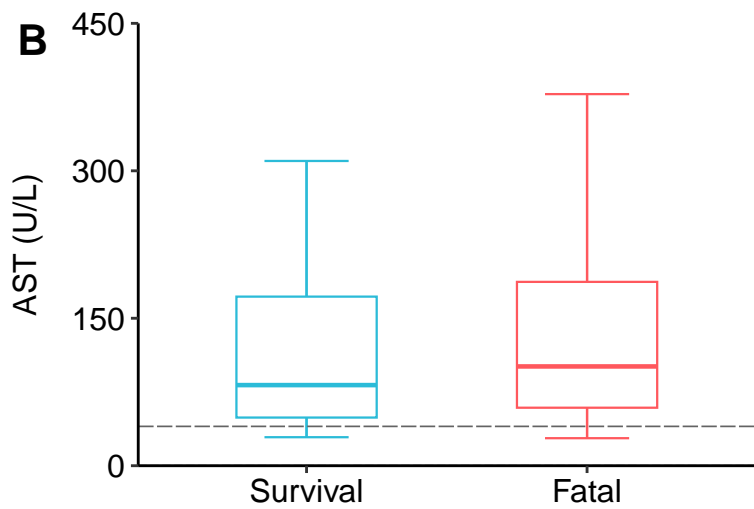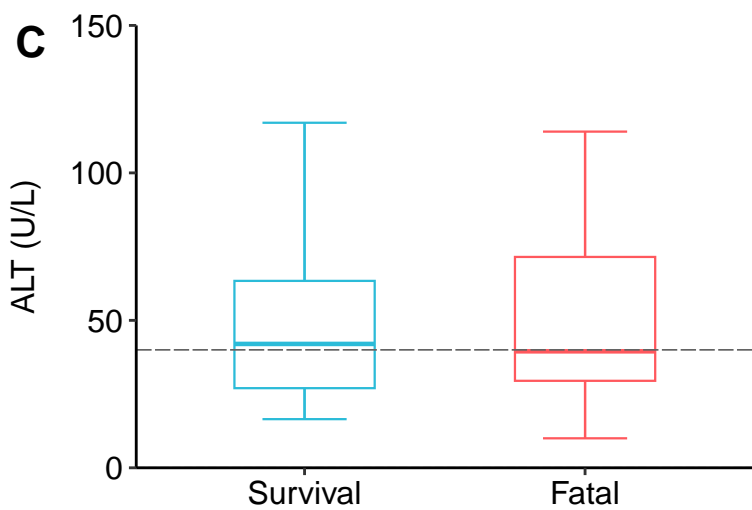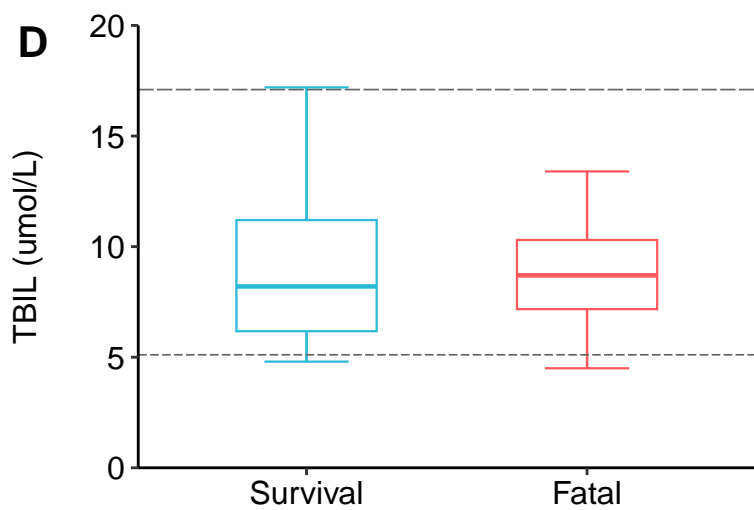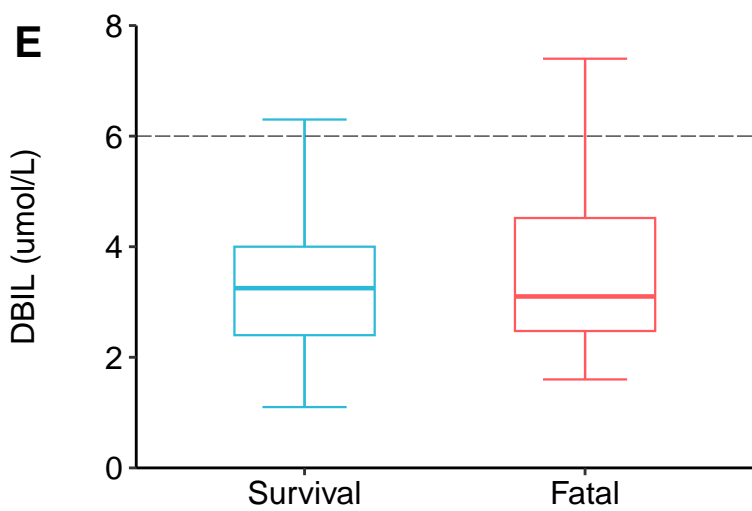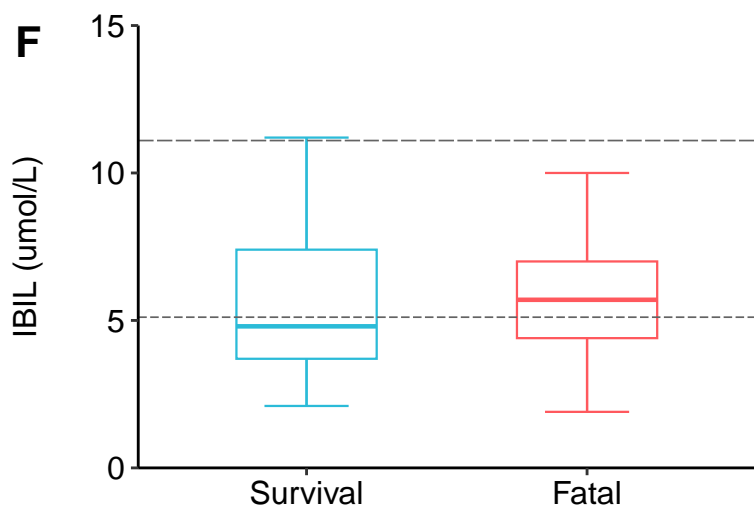

Supplement: Supplementary file 7 — Additional file 7: Fig. S7. The comparison of biochemical index level between the case and control groups. A, LDH, lactate dehydrogenase. B, AST, aspartate transaminase. C, ALT, alanine transaminase. D, TBil, total bilirubin. E, DBil, direct bilirubin. F, IBil, indirect bilirubin. The lines in the middle and the top and bottom borders of the box represented the median and the upper and lower quartiles respectively. The top and bottom error bars represented the maximum and minimum respectively [file 12985_2024_2285_MOESM7_ESM.pdf]

Survival Fatal

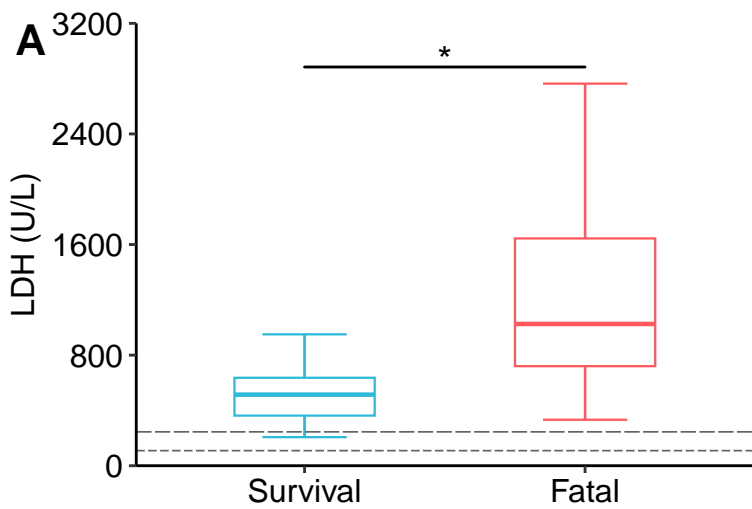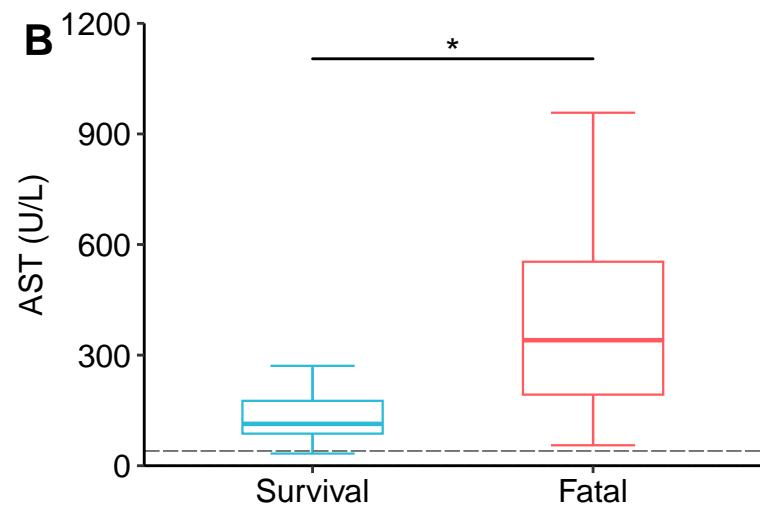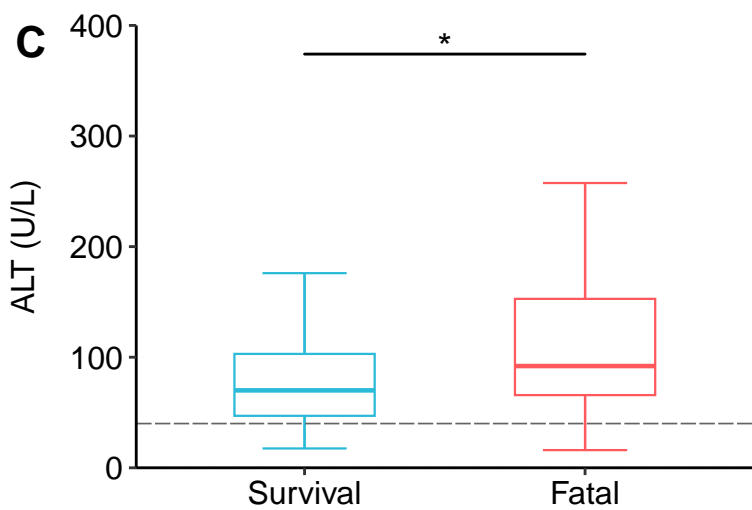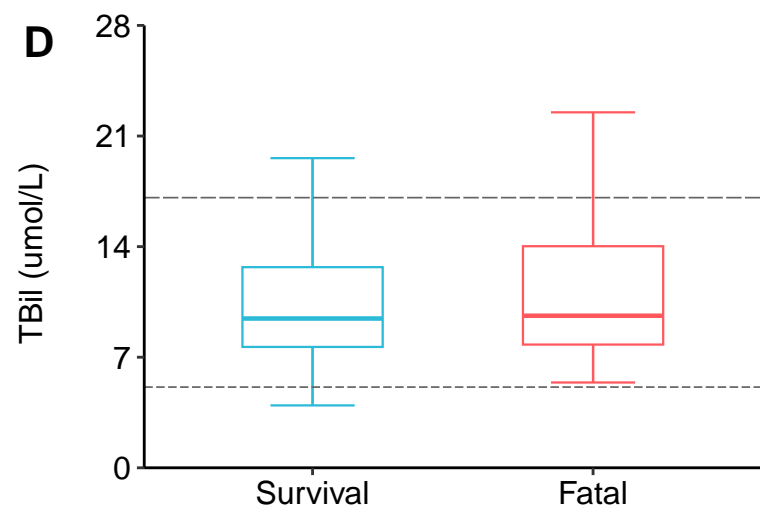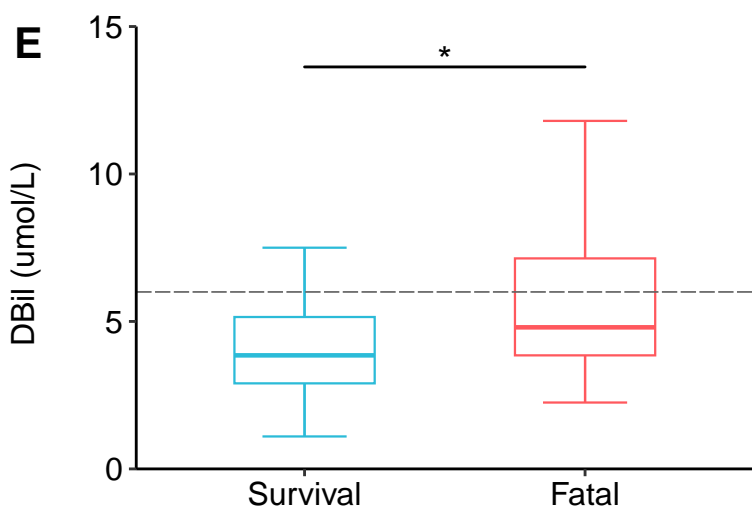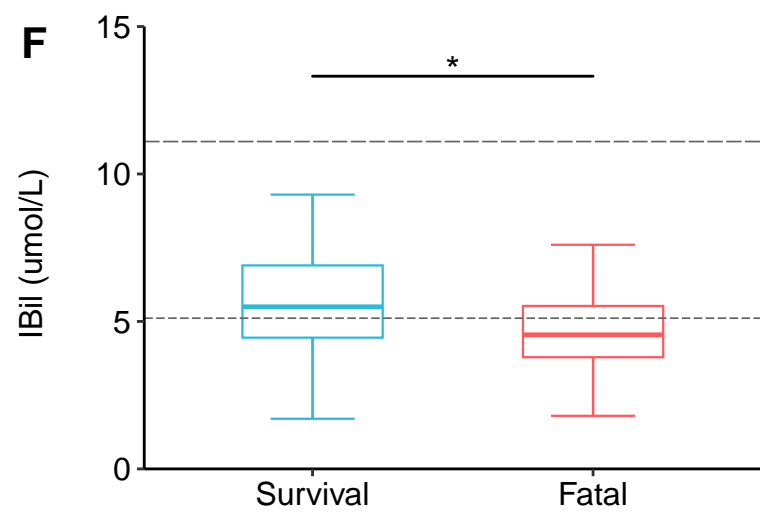

Supplement: Supplementary file 8 — Additional file 8: Fig. S8. The comparison of biochemical index level between the fatal and survival groups. A, LDH, lactate dehydrogenase. B, AST, aspartate transaminase. C, ALT, alanine transaminase. D, TBil, total bilirubin. E, DBil, direct bilirubin. F, IBil, indirect bilirubin. The lines in the middle and the top and bottom borders of the box represented the median and the upper and lower quartiles respectively. The top and bottom error bars represented the maximum and minimum respectively [file 12985_2024_2285_MOESM8_ESM.pdf]
