# Supplementary material for: Metabolic alterations in urine among the patients with severe fever with thrombocytopenia syndrome
Source: Virol J. 2024 Jan 8;21:11. doi: 10.1186/s12985-024-02285-2 (PMC10775654; doi:10.1186/s12985-024-02285-2)
Supplement: Supplementary file 9 — Additional file 9: Supplementary methods and Table S1-S6. The detailed methods for quasi-targeted metabolomics of serum samples were described at the beginning, followed by Table S1-S6 which showed the list of hematologic indexes obtained in the laboratory tests, the concentration of differential metabolites between the control and case groups, the concentration of differential metabolites between the survival and fatal groups, the concentration of differential metabolites between the control and fatal groups, the concentration of differential metabolites between the control and survival groups, and the usage of compound amino acid injection in the survival and fatal groups. [file 12985_2024_2285_MOESM9_ESM.docx]

## Quasi-targeted metabolomics for serum

The samples (100 μL) were placed in the EP tubes and resuspended using prechilled 80% methanol and 0.1% formic acid by well vortex. These samples were then incubated on ice for 5 min and centrifuged for 20 min at 15,000 g, 4 °C. Some of supernatant was diluted to final concentration containing 53% methanol by LC-MS grade water. Subsequently, the samples were transferred to a fresh Eppendorf tube and then were centrifuged at 15,000 g, 4 °C for 20 min. Finally, the supernatant was injected into the LC-MS/MS system analysis ^1,2^. LC-MS/MS analyses were conducted with an ExionLC™ AD system coupled with a QTRAP^®^ 6500+ mass spectrometer. Samples were then injected onto a Xselect HSS T3 (2.1×150 mm, 2.5 μm) through a 20-min linear gradient at a flow rate of 0.4 mL/min for the positive/negative polarity mode. The eluents were eluent A (0.1% Formic acid-water) and eluent B (0.1% Formic acid-acetonitrile) ^3^. The solvent gradient was set as the following: 2% B, 2 min; 2-100% B, 15.0 min; 100% B, 17.0 min; 100-2% B, 17.1 min; 2% B, 20 min. QTRAP^®^ 6500+ mass spectrometer was operated in positive polarity mode with Curtain Gas of 35 psi, Collision Gas of Medium, IonSpray Voltage of 5500V, Temperature of 550 °C, Ion Source Gas of 1:60, Ion Source Gas of 2:60. QTRAP^®^ 6500+ mass spectrometer was operated in negative polarity mode with Curtain Gas of 35 psi, Collision Gas of Medium, IonSpray Voltage of -4500V, Temperature of 550 °C, Ion Source Gas of 1:60, Ion Source Gas of 2:60. The detection of the experimental samples using Multiple Reaction Monitoring were based on in-house database. The Q3 were used for metabolite quantification. The Q1, Q3, retention time, declustering potential and collision energy were used to the metabolite identification. The data files generated by HPLC-MS/MS were processed using the SCIEX OS Version 1.4 to integrate and correct the peak. The main parameters were set as follows: minimum peak height, 500; signal/noise ratio, 5; gaussian smooth width, 1. The area of each peak represented the relative content of the corresponding substance.

**References**

1. Want EJ, O'Maille G, Smith CA, et al. Solvent-dependent metabolite distribution, clustering, and protein extraction for serum profiling with mass spectrometry. *Anal Chem* 2006; **78**(3): 743-52.

2. Barri T, Dragsted LO. UPLC-ESI-QTOF/MS and multivariate data analysis for blood plasma and serum metabolomics: effect of experimental artefacts and anticoagulant. *Anal Chim Acta* 2013; **768**: 118-28.

3. Luo P, Dai W, Yin P, et al. Multiple reaction monitoring-ion pair finder: a systematic approach to transform nontargeted mode to pseudotargeted mode for metabolomics study based on liquid chromatography-mass spectrometry. *Anal Chem* 2015; **87**(10): 5050-5.

## Supplementary Table 1. The list of hematologic indexes obtained in the laboratory tests

| Indexes | Abbreviation | Normal range | Unit |
| --- | --- | --- | --- |
| Lactate dehydrogenase | LDH | 109-245 | U/L |
| Aspartate transaminase | AST | 0-40 | U/L |
| Alanine transaminase | ALT | 0-40 | U/L |
| Total bilirubin | TBil | 5.11-17.10 | umol/L |
| Direct bilirubin | DBil | 0-6 | umol/L |
| Indirect bilirubin | IBil | 5.11-11.10 | umol/L |

## Supplementary Table 2. The concentration of differential metabolites between the control and case groups

| Metabolites | Concentration (peak area), median (IQR) | | P |
| --- | --- | --- | --- |
|  | Control | Case |  |
| 5-Hydroxyindoleacetate | 89085.90 (5708.71-365589.24) | 8270171.89 (3829418.65-18732090.33) | <0.001 |
| 5-Hydroxy-L-Tryptophan | 980846.24 (765568.79-1658848.20) | 7207252.03 (4919639.34-10353100.65) | <0.001 |
| 3-Hydroxyanthranilic acid | 315698.06 (241424.93-419284.01) | 582585.00 (358118.40-1451310.54) | <0.001 |
| L-Kynurenine | 783.74 (783.74-52265.19) | 2964389.74 (1318648.52-6587411.44) | <0.001 |
| 5-Methoxyindoleacetate | 2020554.00 (308119.63-6063181.74) | 395790.01 (181483.80-1077253.63) | 0.035 |
| Theobromine | 14195257.97 (5237804.14-28822367.53) | 3537324.62 (607330.42-7798125.54) | <0.001 |
| 3,7-Dimethyluric acid | 1117318.62 (543365.55-3482577.15) | 99877.55 (4929.54-418981.26) | <0.001 |
| Phenylpyruvic acid | 193793.33 (147925.64-271045.80) | 997900.68 (578797.52-1969648.14) | <0.001 |
| Hippuric acid | 92619056.18 (40223285.41-312403514.05) | 37874893.61 (14208572.19-84519182.53) | 0.002 |
| D-Ribose | 627110.49 (497267.21-861052.20) | 1283064.82 (967349.29-1572325.49) | <0.001 |
| D-Gluconic acid | 30065336.95 (23893934.96-41288030.08) | 76632102.28 (52791777.48-459304215.88) | <0.001 |
| Gluconolactone | 1277034.91 (840842.10-1757247.77) | 1859761.72 (1398155.05-2357740.89) | 0.002 |

## Supplementary Table 3. The concentration of differential metabolites between the survival and fatal groups

| Metabolites | Concentration (peak area), median (IQR) | | P |
| --- | --- | --- | --- |
|  | Survival | Fatal |  |
| Phenylpyruvic Acid | 630852.01 (364236.58-1254091.94) | 1632825.54 (838059.15-2464003.51) | 0.002 |
| Hippuric acid | 23735576.87 (8525885.85-45825008.46) | 49705865.90 (23545721.18-100216898.08) | 0.028 |
| 5-Hydroxyindoleacetate | 4063010.60 (2264801.79-10946169.82) | 12482112.82 (7001608.48-22693959.35) | 0.002 |
| 5-Hydroxy-L-Tryptophan | 5714209.61 (3563753.61-8087432.52) | 8598115.81 (5858558.55-11394686.10) | 0.014 |
| L-Kynurenine | 1447322.31 (854604.07-3973170.20) | 4628250.20 (2572126.88-8312684.45) | 0.004 |
| Sucrose | 8798481.27 (3730218.81-15907321.10) | 20345047.25 (10538243.46-30164452.71) | 0.016 |
| Trehalose | 5379196.60 (2918543.63-10752316.73) | 12760581.62 (7697524.69-22416430.29) | 0.013 |

## Supplementary Table 4. The concentration of differential metabolites between the control and fatal groups

| Metabolites | Concentration (peak area), median (IQR) | | P |
| --- | --- | --- | --- |
|  | Control | Fatal |  |
| 5-Hydroxy-L-tryptophan | 2296676.09 (1935392.65-3953499.05) | 17528739.69 (12271608.08-22094272.24) | <0.001 |
| 5-Hydroxyindoleacetate | 89085.90 (5708.71-365589.24) | 12482112.82 (7001608.48-22693959.35) | <0.001 |
| 3-Hydroxyanthranilic acid | 315698.06 (241424.93-419284.01) | 518037.61 (333357.54-852176.11) | <0.001 |
| L-Kynurenine | 783.74 (783.74-52265.19) | 4628250.20 (2572126.88-8312684.45) | <0.001 |
| Indoleacetic acid | 1831450.03 (842116.90-3701311.70) | 872505.55 (509545.98-1452547.84) | 0.015 |

## Supplementary Table 5. The concentration of differential metabolites between the control and survival groups

| Metabolites | Concentration (peak area), median (IQR) | | P |
| --- | --- | --- | --- |
|  | Control | Survival |  |
| 5-Hydroxyindoleacetate | 89085.90 (5708.71-365589.24) | 4063010.60 (2264801.79-10946169.82) | <0.001 |
| 5-Hydroxy-L-tryptophan | 2296676.09 (1935392.65-3953499.05) | 12152701.94 (7935563.00-16503663.21) | <0.001 |
| 3-Hydroxyanthranilic acid | 315698.06 (241424.93-419284.01) | 894546.81 (407922.10-2276228.27) | <0.001 |
| L-Kynurenine | 783.74 (783.74-52265.19) | 1447322.31 (854604.07-3973170.20) | <0.001 |
| 5-Methoxyindoleacetate | 2020554.00 (308119.63-6063181.74) | 392147.03 (178475.36-958186.97) | 0.039 |

## Supplementary Table 6. The usage of compound amino acid injection in the survival and fatal groups

| Group | Median dose (mL/day) | Lower quartile  (mL/day) | Upper quartile  (mL/day) | P |  |
| --- | --- | --- | --- | --- | --- |
|  | |  |  |  | 0.751 |
| **Survival** | | 250 | 200 | 250 |  |
| **Fatal** | | 250 | 200 | 250 |  |
